# Bat selfies: photographic surveys of flying bats

**DOI:** 10.1007/s42991-022-00233-7

**Published:** 2022-04-07

**Authors:** Jens Rydell, Danilo Russo, Price Sewell, Ernest C. J. Seamark, Charles M. Francis, Sherri L. Fenton, M. Brock Fenton

**Affiliations:** 1 Department of Biology, Lund University, 22362 Lund, Sweden; 2 Wildlife Research Unit, Dipartimento di Agraria, Università degli Studi di Napoli Federico II, via Università 100, Portici, 80055 Naples, Italy; 3 Copperhead Environmental Consulting, 471 Main Street, Richmond, KY USA; 4 AfricanBats NPC, 357 Botha Ave, Kloofsig, 0157 South Africa; 5 Canadian Wildlife Service, Environment and Climate Change Canada, Ottawa, ON Canada; 6 Department of Biology, University of Western Ontario, London, ON Canada

**Keywords:** Bat identification, Conservation, High-speed photography, Monitoring, Species recognition

## Abstract

The recent pandemic and other environmental concerns have resulted in restrictions on research and surveys involving capture and handling bats. While acoustic surveys have been widely used as an alternative survey method, in this study, we show how photographic surveys can offer an important contribution to study and survey bats. We outline approaches, using high speed flash and automated trip beams to obtain photos of flying bats of sufficient quality for reliable identification of species. We show, through a series of examples of setups and photographs, that photography is effective for surveying bats at a variety of sites, where bats roost, drink, and forage. We note, however, that photographic surveys cannot replace capture in all situations. In addition, although photographing bats is less invasive than capturing them, it can involve disturbance, so we stress the importance of minimizing the impact of such operations on bats.

**Supplementary Information:**

The online version contains supplementary material available at 10.1007/s42991-022-00233-7.

## Introduction

Traditional methods for surveying bats often involve capturing and handling them, using a variety of methods such as mist nets, hand nets or harp traps, but there are many situations where this may have adverse impacts on the bats. Disturbance due to capture can cause bats to abandon sites where they roost, forage or drink (Adams and Hayes 2021) and could lead to declines in populations (Law and Blakey 2021). Contact between humans and bats also carries the risk of transferring diseases among bats within a site, among bats in different locations, including different countries or even continents (e.g., White Nose Syndrome Frick et al. 2010; Davy et al. 2020), or between humans and bats. The COVID-19 pandemic has heightened awareness of the risk of human-bat transfer of diseases, resulting in restrictions to research involving capturing and handling bats (Melber et al. 2020).

The most widely used non-invasive approach for surveying bats involves acoustic methods (e.g., Jones and Holderied 2007; Rydell et al. 2017; Zamora-Gutierrez et al. 2021). They have revolutionized the study of bats (Parsons and Szewczak 2009; Fraser et al. 2020), as many species of bats can be accurately identified by their echolocation calls. However, acoustic surveys also have their limitations. For instance, in large colonies or other areas where bats fly in confined spaces, the echolocation calls from different individuals or species may overlap, complicating identification, and potentially resulting in some species going undetected. Furthermore, some species are difficult to identify reliably from echolocation calls alone, particularly as the structure of their calls may change according to habitat structure (Russo et al. 2018). Finally, many species use very low intensity echolocation calls (‘whispering bats’; Griffin 1959), that can only be detected if the bat is very close to the microphone, while most pteropodid fruit bats do not echolocate at all.

Photographic surveys present an alternative non-contact, low-impact way to survey bats (Altenbach and Dalton 2009; Rydell and Russo 2014), but they have been relatively little used for these species. Camera traps are widely and increasingly used for study of other species of mammals (O’Connell et al. 2011; Trolliet et al. 2014; Steenweg et al. 2017). The first camera traps for mammals were deployed as early as the late 19th and early twentieth centuries, using trip wires and other mechanical approaches to trigger a camera (Kucera and Barrett 2011). Their use greatly expanded with the advent of commercially available small, robust and portable camera traps, especially those using digital cameras, and they are now one of the primary tools for surveying many species of mammals (Trolliet et al. 2014). In many cases, they can be used not only for determining the presence of species, but also recognizing individuals, estimating population size or density, and studying behavior (O’Connell et al. 2011; Burton et al. 2015; Karczmarski et al. 2022a, b). Nevertheless, little attention has been paid to their potential for working with bats, and recent reviews on the use of camera traps for wildlife studies did not even mention bats (Trolliet et al. 2014; Burton et al. 2015; Steenweg et al. 2017). This may be largely because bats present some special challenges for photographic surveys. Accurate identification of bats to species in flight usually requires close-up sharply focused photographs, which can be challenging to obtain for small, fast-moving, flying nocturnal animals. Conventional camera traps used for terrestrial mammals are often too slow or insensitive to detect flying bats, and if they do capture a photograph of a bat, the photo is rarely sharp enough or close enough to allow species identification.

**Fig. 1﻿:**
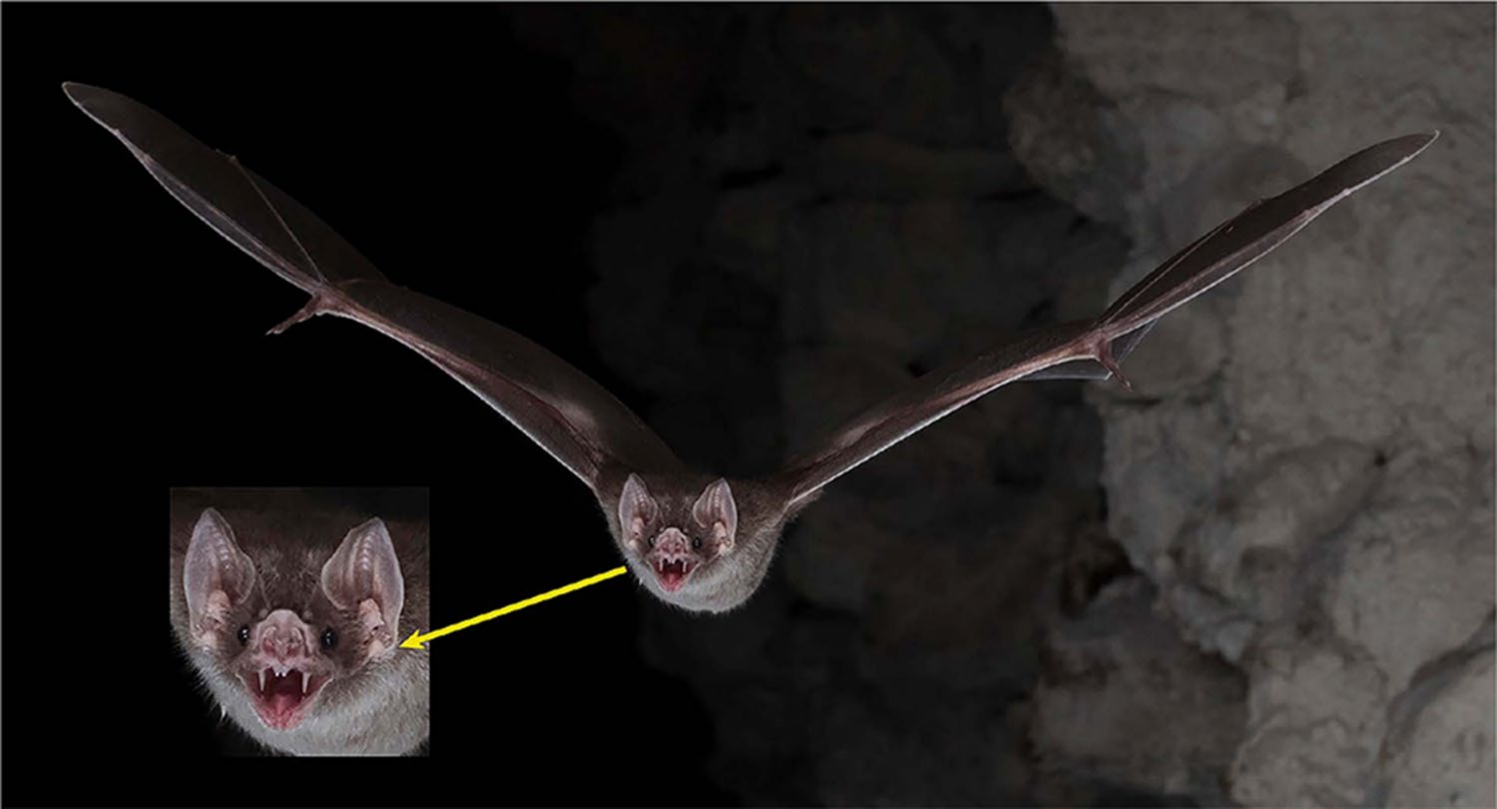
A common vampire bat (*Desmodus rotundus*) flying directly at the camera presents distinctive facial and dental features (arrow)*.* The bat was emerging from a tunnel roost in Belize. Nikon D850, 50 mm Nikon lens, f/16, bulb, ISO 400. Photo by S. L. Fenton and M. B. Fenton

**Fig. 2:**
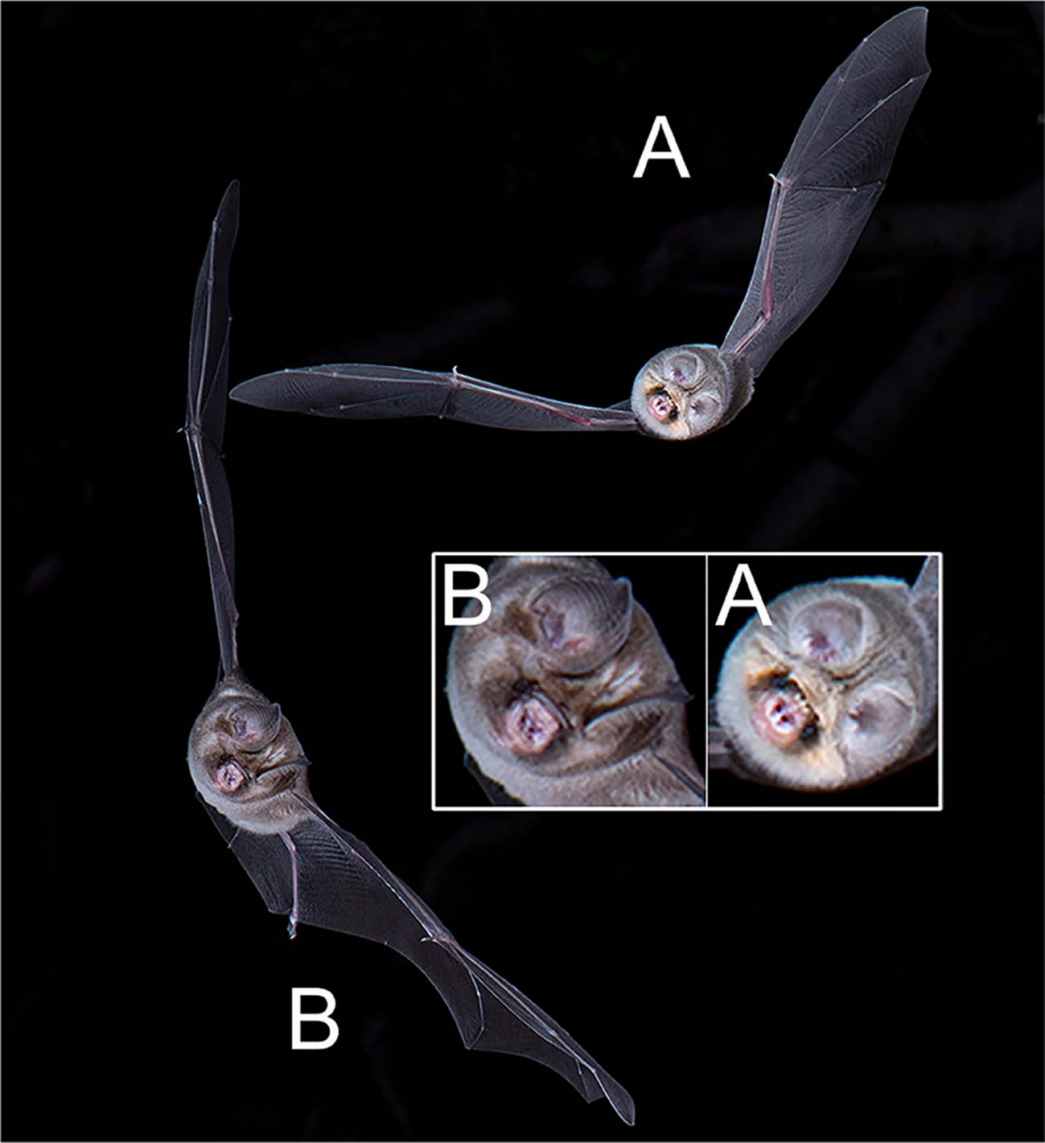
A Percival’s trident bat (*Cloeotis percivali*, **A**) and a Sundevall’s roundleaf bat (*Hipposideros caffer*, **B)** belong to two families, Rhinonycteridae and Hipposideridae, respectively. Note the differences in facial details. In *Cloeotis* the noseleaf has three prominent bumps on the top, forming a trident, while the upper part of the noseleaf is smooth in *Hipposideros*. This is an example of a situation in which the bats are morphologically distinct and also readily distinguished by the dominant constant frequency components of their echolocation calls, ~ 142 kHz in Sundevall’s roundleaf bat and ~ 212 kHz in Percival’s trident bat. The bats were flying in a wooded area around a cave entrance in South Africa. Nikon D850, Nikon 105 mm macro, f/18, bulb, ISO 125. Photo by S. L. Fenton and M. B. Fenton

**Fig. 3:**
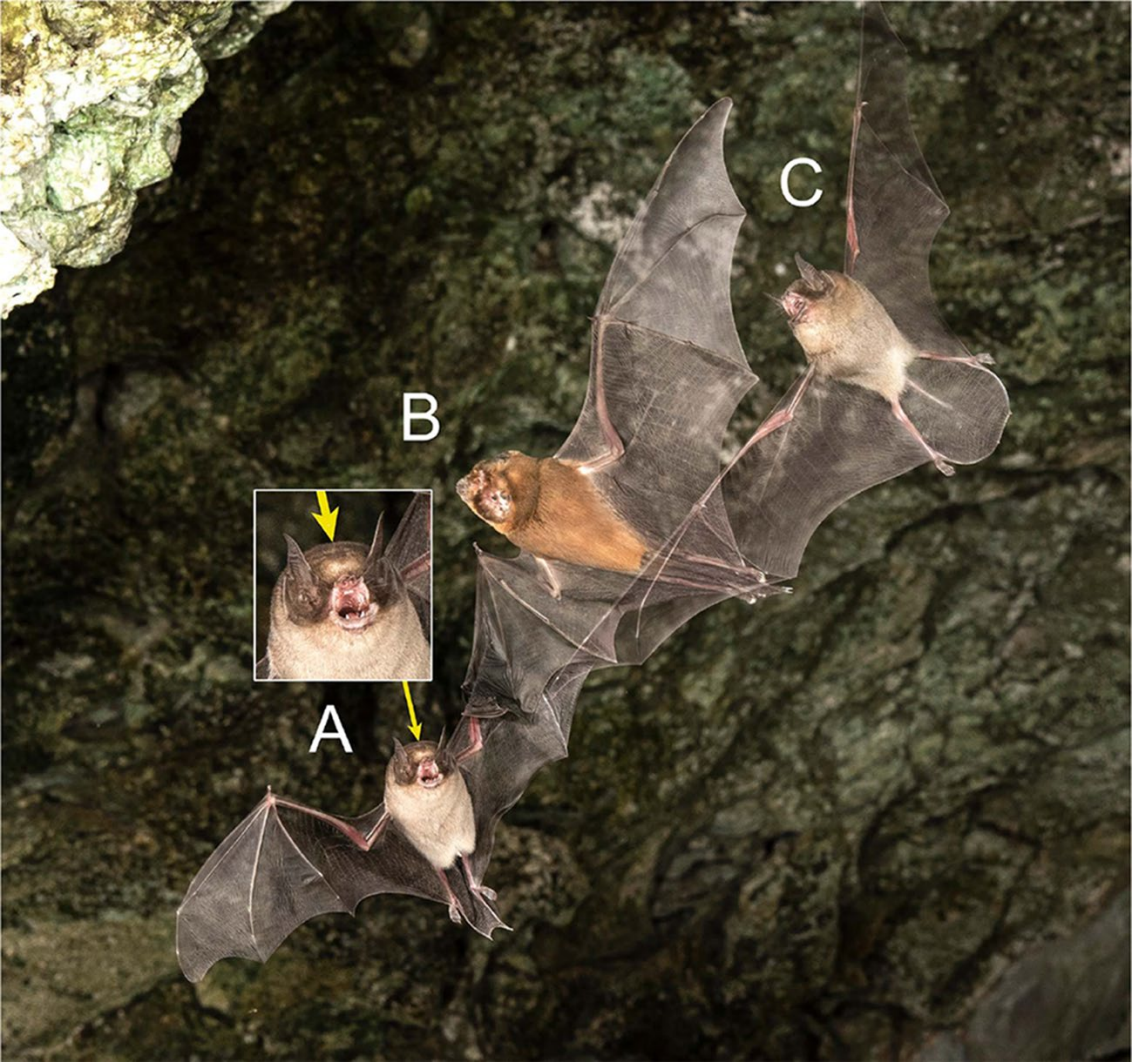
Two species of moustached bats (*Pteronotus*, **A**, **C**) flying with an Antillean ghost-faced bat (*Mormoops blainvillei*, **B**) in a cave in Jamaica. The *Mormoops* differs from the *Pteronotus* in color and facial appearance. Parnell’s moustached bat (*Pteronotus parnellii*, **A**) has a distinctive bump on top of its muzzle (arrow), a feature lacking in Macleay’s moustached bat (*Pteronotus macleaya*, **C**). In this photo, each bat tripped the beam separately on a single exposure, producing a “ghosting” effect. Nikon D810, 60 mm Nikon macro lens, f/16, bulb, ISO 400. Photo by S. L. Fenton and M. B. Fenton

**Fig. 4:**
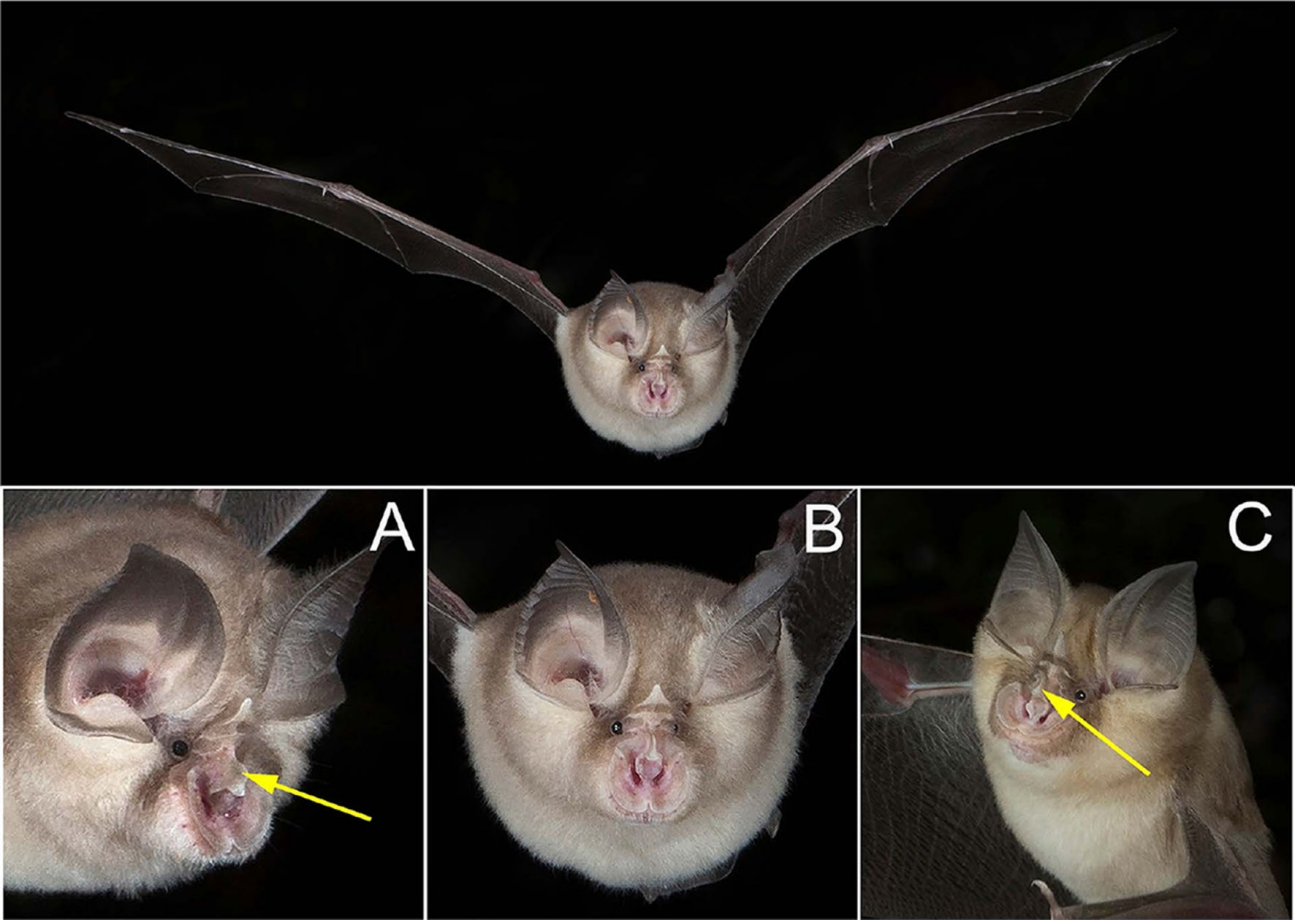
Close-ups of the faces of two species of horseshoe bats (*Rhinolophus*) photographed as they flew near the entrance to a cave in South Africa. In Bushveld horseshoe bats (*Rhinolophus simulator*, **A**) the connecting process (arrow) is a smooth curve (arrow), obvious in lateral view. In Blasius’ horseshoe bat (*Rhinolophus blasii*, **C**) the connecting process (arrow) is acutely pointed. The shape of the connecting process and hence the species cannot be reliable determined in a full frontal view even when zoomed in **B**. In this situation, a concurrent bat detector could have helped identify the bats, as most energy in the CF part of the echolocation call is ~ 87 kHz in Blasius’ horseshoe bat, and ~ 82 kHz in bushveld horseshoe bats. Bushveld horseshoe bat (**A**) photographed with Nikon D810, 60 mm macro, f/18, ISO 200; Blasius’ horseshoe bat (**B**) with Nikon D850 with 50 mm lens, f/16, ISO 150. Unidentified *Rhinolophus* (**C**) with Nikon D810, 60 mm lens, f/18, ISO 200. Photos by S. L. Fenton and M. B. Fenton

The objective of our paper is to describe approaches for taking high quality photographs of bats in natural settings that can be used for non-invasive surveys of bats, both to determine which species may be in an area and to learn more about their natural behavior. This paper expands upon the approaches described in Rydell and Russo (2014), providing details on the types of equipment that are most effective and some of the different ways they can be deployed to get clear, identifiable photos. To illustrate the potential of the approach, we present a number of examples, both in the text and in supplemental materials, selected from a collection of photographs taken by the various authors that includes over 56 species of bats. Through these examples, we also highlight some of the features that need to be captured in the photographs to identify bats to species. While it may be relatively ‘easy’ to obtain a picture of a flying bat, it is more challenging to get sharp, well-exposed images that show crucial details for identification (e.g., Figs. 1, 2, 3, 4).

## Methods

The overall approach for photographing bats in flight involves using a “camera trap” consisting of one or more high-resolution cameras with good quality lenses, high-speed flashes and a beam trigger system that activates the cameras and flashes when tripped by a flying bat.

### Cameras

A variety of different types of cameras can be used as long as they have the following features: (1) have a high-resolution sensor; (2) have the option to operate on a ‘bulb’ setting with manual opening and closing of the shutter; (3) can be triggered via a cable or an electronic shutter release; (4) allow for adjustable aperture settings (f-stops) to get good depth of field; (5) allow for manual exposure control; (6) allow for manual focus; and (7) are compatible with the preferred lenses and flashes. We typically use digital single lens reflex (DSLR) or mirrorless cameras combined with appropriate lenses. We prefer full frame cameras with a 35 mm sensor, as these typically have larger, more sensitive pixels that deliver less grain/noise at a given ISO. However, good quality photographs can also be obtained with smaller sensors, especially with newer cameras. High resolution sensors (20–30 megapixels or higher) provide greater flexibility in cropping images given the bats often do not fill the frame, as well as allowing enlargement to see small details more clearly.

### Lenses

The choice of lens should reflect the size of the bats anticipated and the proximity of the camera to the position where the bats will trip the beam. Ideally, a lens should be selected so that the bat largely fills the frame of the camera, to ensure the highest resolution possible. However, there is a trade-off between the size of the bat in the frame, and the likelihood that the whole bat will be captured in the photo. It can be quite frustrating to get a sharp full-frame photo of a bat where its head or feet or wing tips are cut-off at the edge of the photograph. Typically, for photographing small bats, we set the camera 0.5–1.5 m away from the focal point (where the bat will trip the beam), using a lens with a focal length of 50–70 mm on a full-frame camera. This presents a compromise, where the bat will typically fill about one quarter to half of the frame, thus increasing the chance it will be fully within the picture. However, in some situations, we have used wide-angle lenses (sometimes as short as 14 mm) if the camera will be very close to the bat, or if the position of the bat cannot be reliably predicted. In other situations, where the camera is positioned farther from the focal point (which works best if the flash is not attached to the camera), we have used telephoto lenses up to 150 mm or more.

Most of us (SLF, MBF; ES; PS; CMF) tend to shoot with more than one camera to increase the odds of getting a clear identifiable photo. Each camera may have a different length lens to get close-ups or wider angles, or be set at a different angle to obtain lateral or frontal views. However, this is a matter of personal preferences. One of us (JR) always used a single camera, one beam and as few accessories as possible to minimize the risk of technical failure and to facilitate carrying and setting up equipment without disturbing the bats.

### Flashes

One or more high-speed flashes are essential to obtain sharp, well-lit photographs of rapidly flying bats that are clear enough to show crucial anatomical features needed for accurate identification of the bats (e.g., Figs. 1, 2, 3, 4). A flash that lasts 1/20,000 of a second is sufficient to “freeze” (prevent motion blur of) a bat flying 5 m s^−1^. To achieve short flash durations, we recommend use of commercially available electronic flashes that can be set for manually adjustable power output—automatic exposures do not allow appropriate control. In many cases, 1/64 of full power works well, but durations in fractions of a second at different powers vary considerably among flashes. According to the specifications in the manuals of six readily available models of flashes, the duration at 1/64 power varied from 1/18,000 s to 1/35,700 s. Some flashes have settings for 1/128 power or even 1/256, which could produce sharp images of even faster moving bats. However, the shorter duration produces less light, which requires other trade-offs, in depth of field or ISO settings, to get an adequate exposure. The zoom setting on the primary flashes should be adjusted to concentrate the light as much as possible, while ensuring that it still fully illuminates a bat at the focal point.

We recommend using several flashes to achieve better balanced lighting, to avoid dark shadows on parts of the bat, to illuminate a larger area, and to increase the overall brightness. All flashes used in a particular set-up should be the same brand and model, and set at the same power level to ensure that they will be perfectly synchronized. Different models of flashes may have different time lags between receiving the trigger signal and reaching full power and may differ in flash duration. Variation in these factors will result in blurred or ghosted images. Depending on the setup, we may use three to eight flashes that are wirelessly synchronized, with one flash (or a separate controller) set as the master to trigger the others using either radio signals or light signals depending on the model.

The distance from the flashes to the focal point is very important, as it determines the brightness and exposure. The light drops off with the inverse of the square of the distance, so doubling the distance from the flash to the focal point results in only one quarter the light reaching the subject. We typically try to set most of the flashes about 0.5–1.0 m from the focal point, to ensure adequate exposure, but this is not always possible depending upon the location. If the flashes are set farther away, this may require reducing the f-stop (and sacrificing some depth of field) or increasing the ISO or both to get an adequate exposure.

### Triggers

**Fig. 5:**
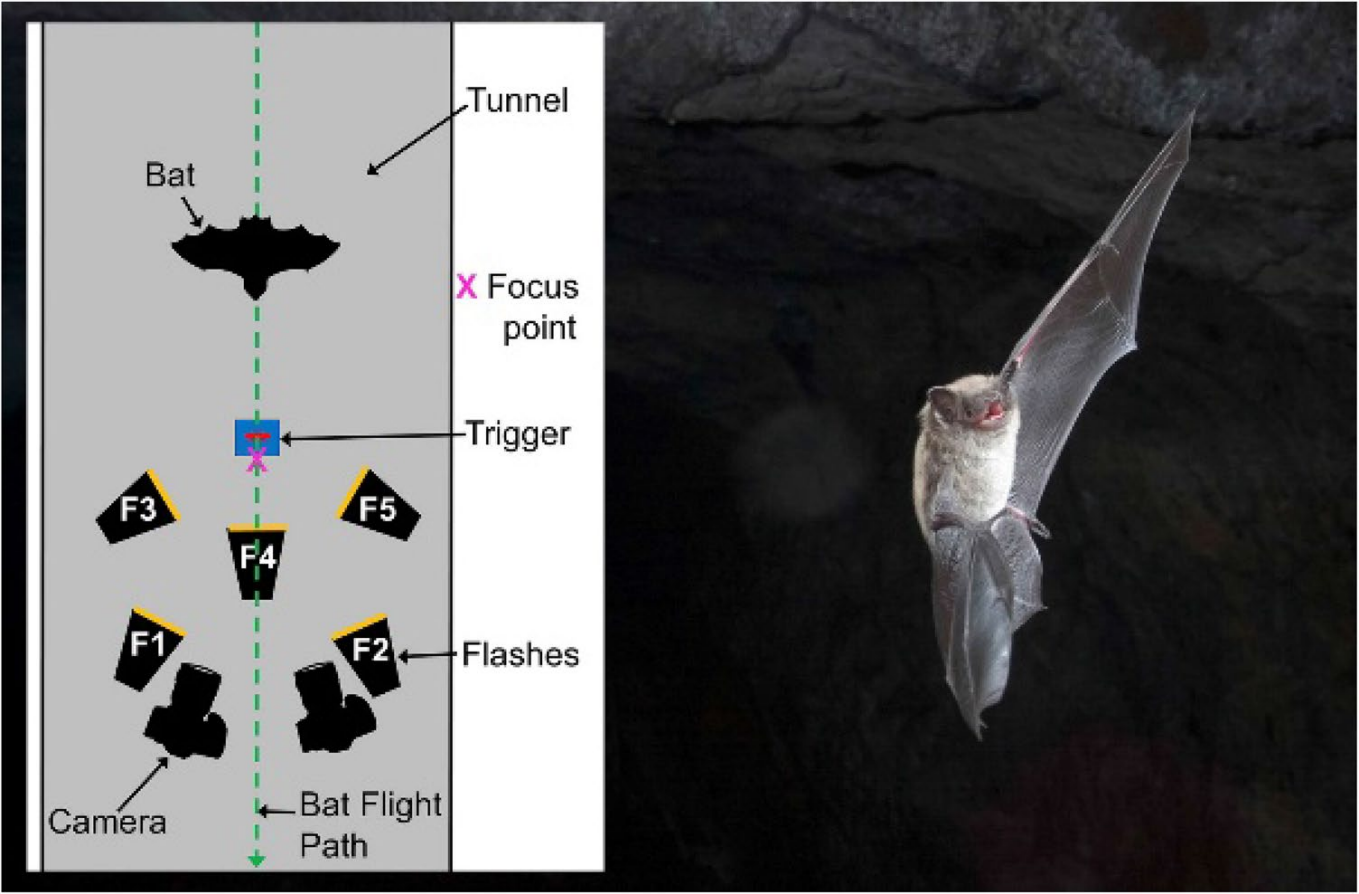
A little brown myotis (*Myotis lucifugus*) emerging from a 2 m high mine tunnel, with a diagram from above showing a typical equipment setup for this type of photograph (not to scale). We set two cameras on tripods about 1 m above the ground, and 0.6–1 m from the focal point, far enough apart to avoid blocking the flight path. Two cameras at different angles increase the chances one will capture diagnostic features of the face or wings. The primary flashes, F1 and F2 are mounted level with each camera, on arms attached to the camera tripods and pointed at the focal point. Two additional flashes (F3, F5) are on light stands aimed down from above the flight path on each side; and one flash (F4) is on a Gorillapod near the ground, illuminating the bat from below. The lack of light in the tunnel meant we could use Bulb (open shutter) setting. The beam trigger points up from below and controls one flash (F4) which triggers the rest remotely. All flashes are set for manual 1/64 power. Camera remote controls are used to open and close the camera shutters. The focus point is close (~ 5 cm) to the trigger to ensure the head is in focus if the body trips the beam, as there is virtually no time lag when using the beam to trigger the flashes. The zoom setting on the flashes was adjusted to achieve the desired balance in lighting. Nikon D1, Nikkor 31 mm lens, f/16, bulb. Photo by M. B. Fenton

**Fig. 6:**
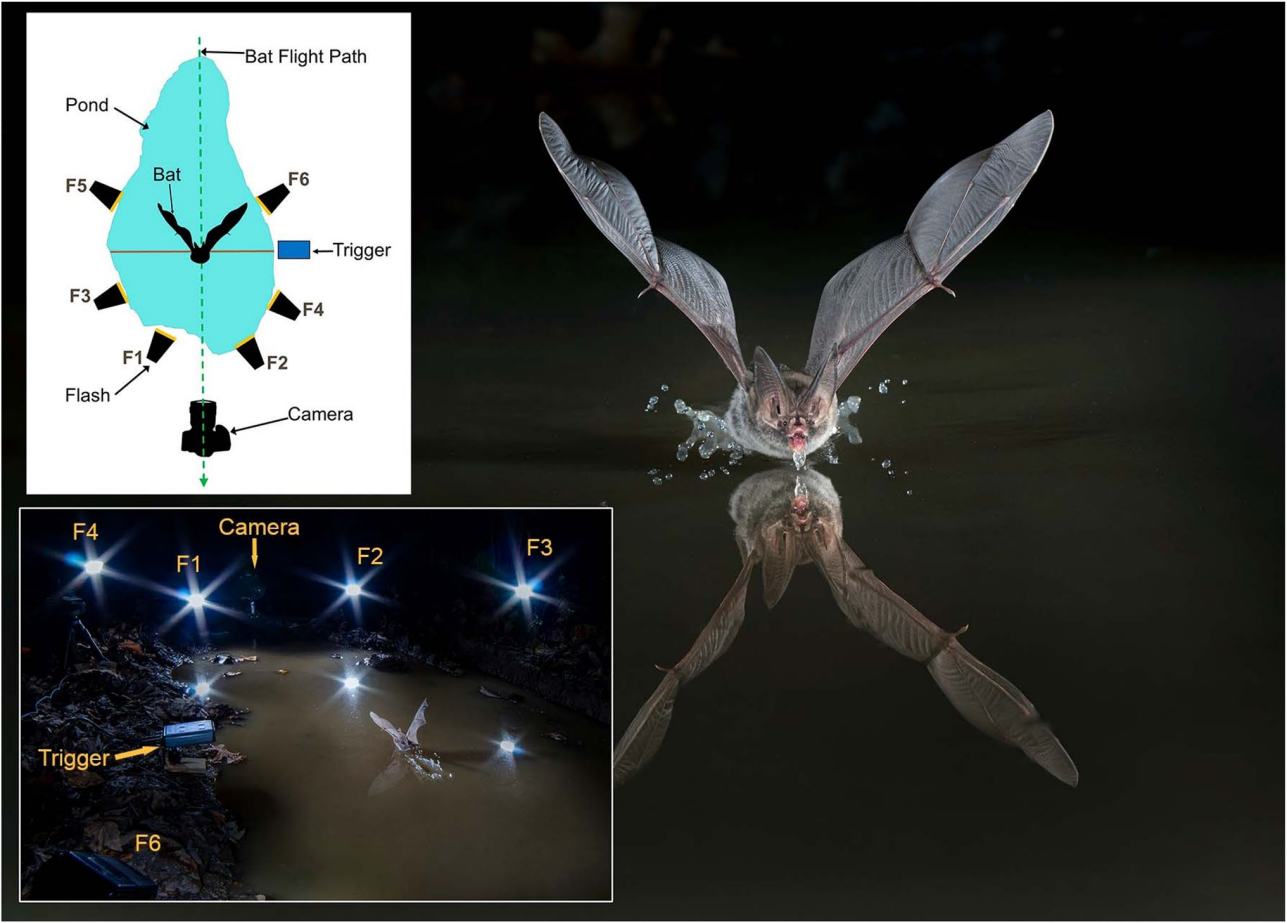
Example of a setup used to photograph bats drinking at a puddle in Kentucky, showing a Rafinesque’s big-eared bat (*Corynorhinus rafinesquii*) flying towards the camera as it drinks (right); a simultaneous photo taken from the opposite end of the pond, showing the bat from behind and the main components of the setup (bottom left); and a diagram of the setup (upper left). Flashes F1 and F2 are aimed at and slightly below the bat, to add a reflection off the water; flashes F3 and F4 are higher on light stands illuminating the bat from above, while flashes F5 and F6 provide backlighting. The camera was set on Bulb with the triggering device (Cognisys Sabre) triggering the flashes and refreshing the camera shutter. The splash from drinking is visible where the bat tripped the beam. This bat is easily identified in this area as the only species with such long ears. Primary photo Nikon D750 with 70–200 mm Tamron lens, f/16, bulb, ISO 400. Photo by P. Sewell

Although it is sometimes possible to trigger the system manually, in most cases it is necessary to use a photoelectric beam to trigger the camera and flashes when a bat flies by the desired location. A variety of high-speed trigger systems are available commercially (see Table 1 for a sample of currently available systems), although somebody with good electronic skills could build their own. The photoelectric switch consists of a transmitter and a sensor, usually combined in a single unit. These typically operate in one of two modes. In one mode, an external reflector is used to reflect emitted light back to the sensor, and the system is triggered when a bat breaks the beam. An angular retroreflector that reflects light back to its source from a wide range of incident angles (similar to reflectors used on bicycles or road signs) is much easier to align than a mirror reflector. In some systems, the transmitter is a separate unit from the receiver and the beam is created by pointing the transmitter at the receiver from the opposite side of the focal point. In the other mode, the bat is the reflector and triggers the system when it reflects the beam back to the sensor. This approach tends to be less sensitive, and hence have a shorter range, because most bats are relatively dark and do not reflect much light back to the sensor except when they are very close. However, this mode can be easier to set up, because it does not require positioning and aligning the reflector on the opposite side of the focal point from the transmitter. Some commercial systems can operate in either mode, while others only have one mode (Table 1). It is possible to use several beams (SLF and MBF have used up to 12) to increase the area sampled and the sensitivity of the system, especially when using the bat as a trigger. In this case, the system is set to trigger when the bat reflects the signal from any beam. Alternatively, some systems allow use of multiple beams to increase the precision of the focal point, by crossing them at the desired focal point, and only triggering the system when all beams are broken simultaneously. In many situations, the sensitivity of the system can be increased by setting the beam to point vertically from underneath where the bats are anticipated to fly, because the bats present a larger target from below than from the side (Fig. 5). However, in other situations, such as when photographing bats flying over water (Fig. 6), the beam needs to be set horizontally.

**Table 1:** Sample of commercially available trigger systems

Trigger system	Manufacturer	Subject as reflector?	Visible beam	Recycle shutter in bulb mode	Keep awake mode for reduced lag when using shutter	Cost May 2021 (USD)^a^	Power	Notes
Sabre	Cognisys	Yes	No	Yes	Yes	$400	Internal rechargeable	Very programmable, long-distance beam
Range IR	Cognisys	Yes	No	No	Optional	$200	2 AA	Stay awake mode requires optional shutter interface switch ($50)
Jokie2	Eltima	Optional^b^	No	No	Yes	$193	4 AA	
Joker2	Eltima	Optional^b^	No	Yes^c^	Yes	$665	3 AA	Includes 3 long distance beams; very programmable
LV5	Cactus	No	Yes	No	No	$110	2 or 4 AAA	Long distance beam. Separate receiver and transmitter
Smart +	Miops	No	Yes	No	No	$240	Internal rechargeable	Requires a separate laser pointer (not included)
Model 33	Phototrap	Optional^b^	Yes	No	No	$460	n/a	May no longer be available

^a^Prices may not include all required cables or other accessories

^b^These devices can be operated either using an external reflector, with the subject breaking the beam, or using the subject as a reflector

^c^Available with software version 2.2.2.0 or later

### Shutter settings

There are two main ways that the system can be operated: with the beam triggering the flashes or with the beam triggering the cameras. In areas with minimal or no ambient light, the camera can be set on the open shutter (Bulb) setting, and the beam set to trigger the flashes when a bat flies by. The advantage of this approach is that there is virtually no time lag between the bat crossing the beam and the flashes firing, which means the bat will still be very close to the preselected focal point when the picture is taken. If multiple cameras are used, they will all capture an image at the same instant, which can be a convenient way to view the bat from different angles. In this situation, the workflow consists of pressing the shutter to open it (preferably with a remote control, either wireless or cabled), waiting until a bat trips the beam to fire the flashes, then closing the shutter again. If multiple cameras are in use, a wired remote control can be set to control all cameras at the same time with a branching cable. Some commercially available electronic trigger systems can automate opening and closing of the shutter, which means the photographer does not need to sit by the system controlling it (Table 1). Automatic control of the shutter also reduces the risk of a double exposure, which can happen if two bats fly by in quick succession, before the photographer closes the shutter (as happened in Fig. 3). Some trigger systems can be set to refresh (close and reopen) the shutter after a fixed time (e.g., 30 s to a few minutes) if no bat flies by, to reduce ghosting caused by any low levels of ambient light or long exposure camera noise (Table 1).

In brighter ambient light, the beam should be set to trigger the camera which, in turn, triggers the flashes. In this case, the shutter speed should be manually set to the shortest duration that the camera supports with flash synchronization to minimize any effect of ambient light. The appropriate flash synchronization speed varies among cameras and needs to be determined by checking the camera manual. In some older cameras, this may be as slow as 1/60 s, but for many newer cameras it is 1/250 s or faster.

The main disadvantage of triggering the camera is that there is a significant delay between the instant when the bat breaks the beam and when the camera takes the picture. This delay varies from about 50 ms to over 250 ms depending on the camera model and whether the camera is awake and fully ready (equivalent to half-pressing the shutter). Some trigger systems have an option to keep the camera constantly awake to minimize the time delay (Table 1). However, even with a 50 ms delay the bat may move some distance between the time it breaks the beam and the time the picture is taken; for example, a bat moving at 5 m s^−1^ will move 0.25 m in 50 ms. When the camera time delay, flight speeds, or flight directions of the bats are not known, one option is to take a few photos using a wide-angle lens (e.g., 15 mm) to determine the location of the bats when the flash is triggered. The lens can then be switched to a longer focal length and focused on the newly determined location to get higher resolution photos of subsequent bats. Another option is to use multiple cameras, each focused on a slightly different location or with a different focal length lens. If multiple cameras are used, they should be set to each trigger the flashes separately, as they are likely to have slightly different time lags. However, if the time lags are too close, but with some variation, some photos may be spoiled if both cameras happen to fire their flashes while the shutter of the other one is open. Some trigger systems allow for triggering each camera separately with a slight time lag if required—it is important to experiment at home to become fully familiar with the system, before setting up in the field.

### Focus and other camera settings

Several settings need to be adjusted to ensure the photographs will be adequately exposed and sharp at the same time, and that the metadata will be accurate.

The focus must always be set to manual, as the autofocus on the camera can never adjust quickly enough for a flying bat. The best way to ensure that the bats will be in focus is to place a sharply contrasting object at the desired focal point during setup. A pole marked with dark and light stripes or a piece of card with a checkerboard pattern can work well for focusing. The pole or other sharply contrasting object should be placed at the focal point, the camera(s) set to autofocus, focused on the pole or other object, then changed back to manual focus, taking care not to touch the focus ring. If the focal object has a known scale (e.g., 1 or 2 cm checkerboard) it can be useful to take one or more pictures of it in the same spot with each camera, to serve as a size scale reference in subsequent photos of bats. As noted above, the relation between the focal point and the position of the beam will depend on whether the beam trips the flash or the camera. Even if the beam trips the flash, which typically has a lag < 1 ms, the focal point may be slightly in front of the beam to ensure the head is in focus if the body is more likely to trip the beam than the head.

We recommend an aperture setting between f/16 and f/32 to provide adequate depth of field so that most of the bat will be in focus, even if it tripped the beam with a wing tip rather than its body. A higher f-stop will produce greater depth of field, but at the cost of lower light, which may require a higher ISO setting. The ISO setting is important because a higher ISO increases sensor sensitivity, but also increases graininess and reduces image quality. Generally, we find that ISO 400-800 allows for adequate exposure at f/16, but higher ISO values may sometimes be necessary, particularly if the flashes are farther away from the focal area. Camera technology is continuously improving, and newer camera models may perform well even at higher ISO.

The clock on the camera should be set to the correct date, time, and time zone (especially worth checking when travelling to a study location). If multiple cameras are used, their clocks should be synchronized as closely as possible so that simultaneous photos of the same bats will have the same time stamp. The white balance should be set for flash photography (typically 5500 K), as auto white balance may not result in the correct setting, especially with ‘bulb’ photography. However, if the camera is set to record raw images, rather than jpg, the white balance and other features such as contrast and to some degree the exposure can also be adjusted during the editing stage.

All power-saving features on the cameras and flashes should be disabled, so that they do not go to sleep or turn off in between bats. Any automated features on the cameras such as red-eye reduction should also be turned off, along with any indicator lights on the flashes or cameras, if possible, to avoid disturbing the bats. If some lights cannot be turned off, it may be possible to cover them with black electrical tape. Cameras that are constantly awake, or set in bulb mode, will use their batteries much more quickly than during normal photography. As a result, all batteries for the cameras and flashes should be fully charged before each session, and sufficient spare batteries and spare memory cards should be available. When travelling to a remote area, it is a good idea to bring spares of any cables or connectors, in case they become damaged.

### System setup

Configuring the system usually requires a variety of different supports to hold the cameras, flashes and beams. Typically, we use sturdy tripods to hold the cameras, and light stands or lightweight tripods supplemented with a variety of commercially available supports such as Magic Arms^®^, Super Clamps^®^, Gorillapods^®^ and Ultrapods^®^ to hold flashes and beams. Some of these supports can be used to fasten multiple items to the same tripod or light stand or even nearby trees or branches. Typically, we might set one flash on each side of the cameras to provide the main lighting, with additional flashes aiming down from above and up from below to ensure the bats are illuminated from all directions. We show two examples of potential setups in Figs. 5 and 6. In practice, compromises are often necessary, especially when setting up in a cave entrance or near a roost with uneven ground or walls. A variety of straps and other fasteners are useful to hold stands securely, so they do not fall over and damage valuable equipment. When positioning the cameras, pay attention to the backgrounds, to ensure that there will not be distracting objects including other equipment or reflected light from the flashes that will affect the photographs. It is a good idea to take a few sample photographs and check the backgrounds and exposure before the bats start to emerge. A small bat-sized brown object (such as a toy bat) can be gently tossed through the beam to ensure that the system works properly and the focus and exposure are adequate. Finally, it is very important to ensure that the supports and other equipment, as well as the photographers, do not block the bats’ flyway, hinder them or make them suspicious of the entire activity.

We strongly recommend practicing the setup at home, to become familiar with all aspects of the equipment including all the camera, flash and trigger settings, and ensure that everything is working. It is much easier to troubleshoot problems at home than in the field.

We also recommend scouting potential sites before setting up, to determine the bats’ behavior, flight paths, timing of emergence and other activities. It is best to start the setup at least a couple of hours before the bats are expected to emerge to have time to test the system and address any problems—setup often takes much longer than anticipated, especially at a new site or in a challenging location. Finally, when waiting for bats, the photographers should plan to be quiet and still, with lights and cell phones turned off, to avoid disturbing the bats.

### Image editing and cropping

In most cases, it is necessary to do some editing and cropping of the resultant images. “Normally” a photographer composes the image during setup, but when the bats take their own photos by tripping a beam, the bats are rarely ideally framed. A variety of programs can be used for editing, such as Adobe Photoshop^®^, Lightroom^®^, GIMP, or DxO Photolab. Images that are saved in camera raw format provide greater flexibility for editing than compressed formats such as jpg, though the photos can be saved in a compressed format after editing. Adjustments may be needed to the white balance, the contrast or the exposure. Most programs allow independent adjustment of exposure in bright and dark areas, which can be used to enhance the contrast between the bat and the background, or bring out details in unwanted shadows. The extent to which the images need to be cropped will depend on where the bat is within the frame, and how well it fills the frame. The sensors of many high-end cameras are > 40,000 pixels, but if the bat is small and the image needs to be cropped 75% in each direction, that would leave only 2500 pixels. While this may be sufficient to fill a high definition screen (1920 × 1080 pixels), it would not allow further enlargement to see details of the face or other features. For this reason, it is desirable to have the bat fill as much of the picture as possible through appropriate selection of lenses.

### Other issues

To minimize disturbance, we recommend not working at the same site more than once per week, especially at small roosts or maternity sites. Tree-roosting species such as western barbastelle (*Barbastella barbastellus*) and common noctule (*Nyctalus noctula*) may abandon roosts even after a single evening of shooting. Disturbance may be less of a problem at swarming sites, night roosts or in large cave entrances. If photographing bats emerging from a roost, all equipment should be removed well before the bats return, to reduce the risk of bats abandoning the roost. Some countries in Europe, including the UK, require a special permit to photograph wild bats at or near a roost.

### Identifying species

**Fig. A1:**
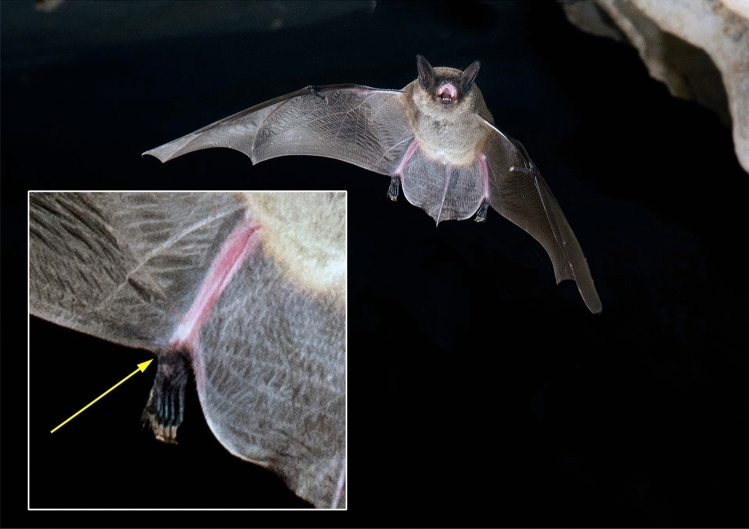
A gray bat (*Myotis grisescens*) swarming at a cave in Kentucky, USA. Inset shows details of the hind foot with the wing attached at the ankle rather than the side of the foot. Three different species of bats were detected at this cave. Nikon D750, 24–120 mm lens at 120 mm, f/16, bulb, ISO 800. Photo by P. Sewell

Many species of bats can be readily identified, at least to family and genus, and often to species, based on the shape of the head and face, noseleaf, ear and tragus or other features, sometimes in combination with fur coloration. For example, common vampire bats (*Desmodus rotundus*) are easily identified by the shape of the face and the teeth (Fig. 1). In a roost occupied by several species, having two or three different species in a frame (Figs. 2, 3, respectively) or multiple separate photos of each species at the same site can facilitate comparison. For our work, we used information about diagnostic morphological features of bats from personal experience and appropriate reference sources for specific sites. Some of the reference books we used included Van Zyll de Jong (1985) for Canada; Barbour and Davis (1969) for the United States; Reid (2009) for Belize; Genoways et al. (2005) for Jamaica; Dietz and Kiefer (2014) for Europe; Monadjem et al. (2010) as well as Patterson and Webala (2012) for south and central Africa; Bates and Harrison (1997) for India; and Payne and Francis (2005) as well as Francis (2019) for SE Asia. We are confident about the identifications to species for at least 54 of the 60+ species we have photographed, although not every photograph of each species can be reliably identified, depending upon the details visible. For example, separation of Blasius’ horseshoe bat (*Rhinolophus blasii*) from bushveld horseshoe bat (*Rhinolophus simulator*) in southern Africa depends on seeing details of the connecting process on the noseleaf, which is visible in some photos but not others (Fig. 4). Some species can only be identified to genus without additional information such as measurements or dental characters. For example, the length of the tibia helps to distinguish Seba’s short-tailed bats (*Carollia perspicillata*) from Sowell’s short-tailed bats (*Carollia sowelli*). Distinguishing Brandt’s myotis (*Myotis brandtii*) from whiskered myotis (*Myotis mystacinus*) or Alcathoe’s myotis (*Myotis alcathoe*) depends upon dental features or penis shape which are not easily seen in photographs. Details such as tragus shape or calcar shape, which help to separate Geoffroy’s bats (*Myotis emarginatus*) from Natterer’s bats (*Myotis nattereri*), or the insertion point of the wing membrane on the foot, which helps identify gray bats (*Myotis grisescens*), are visible in some photographs but not others (e.g., Fig. A1 in Appendix).

In some cases, in the process of identifying bats, we have learned about new diagnostic characters that were not clear in the literature. For example, in Jamaica our initial search of the literature did not list any reliable characters to distinguish brown flower bats (*Erophylla sezekorni*) from Jamaican flower bats (*Phyllonycteris aphylla*). However, we found several diagnostic features through studying our photographs and further comparison with reference material (Fig. S1 in Supplementary Material 1). Some features such as fur color may vary geographically within a species, but can be useful at a particular site to help separate species in photographs. Additional examples of identification characters visible in photographs are presented in Figs. S10, S12, S13, S14 in Supplementary Material 1.

### Combining photography with echolocation

Having a bat detector recording the echolocation calls of bats while photographing them can increase the accuracy of species identification in photos and provide new insights into echolocation calls. This requires ensuring that the clocks on the camera(s) and bat detector(s) are synchronized so the calls can be matched to the photographs. Echolocation calls can be particularly valuable to help identify photos for bats whose distinctive echolocation calls are dominated by one frequency (so-called constant frequency bats), such as species of Rhinolophidae, Rhinonycteridae, Hipposideridae, and some species of *Pteronotus* (Figs. 2, 3, 4; Webala et al. 2019), but can also be helpful for other species. For example, the cryptic common pipistrelle (*Pipistrellus pipistrellus*) and the soprano pipistrelle (*Pipistrellus pygmaeus*), are not easily distinguished in photographs, but use strikingly different echolocation calls (Racey et al. 2009). In contrast, soprano pipistrelles could be confused with Schreiber’s bats (*Miniopterus schreibersii*) based on echolocation calls alone, but in appearance they are quite different. In some cases, photographic identification can be clearer than that based on echolocation calls. For instance, Bechstein’s bat (*Myotis bechsteinii*) is readily identified in a clear photo, but its echolocation calls are difficult to distinguish from those of other sympatric *Myotis* species. In other instances, when the species in the photograph can be reliably identified, this approach can provide new insights into the echolocation calls of the species, including for species whose echolocation calls may not have been studied.

### Other features that can be studied with photographs

**Fig. 7:**
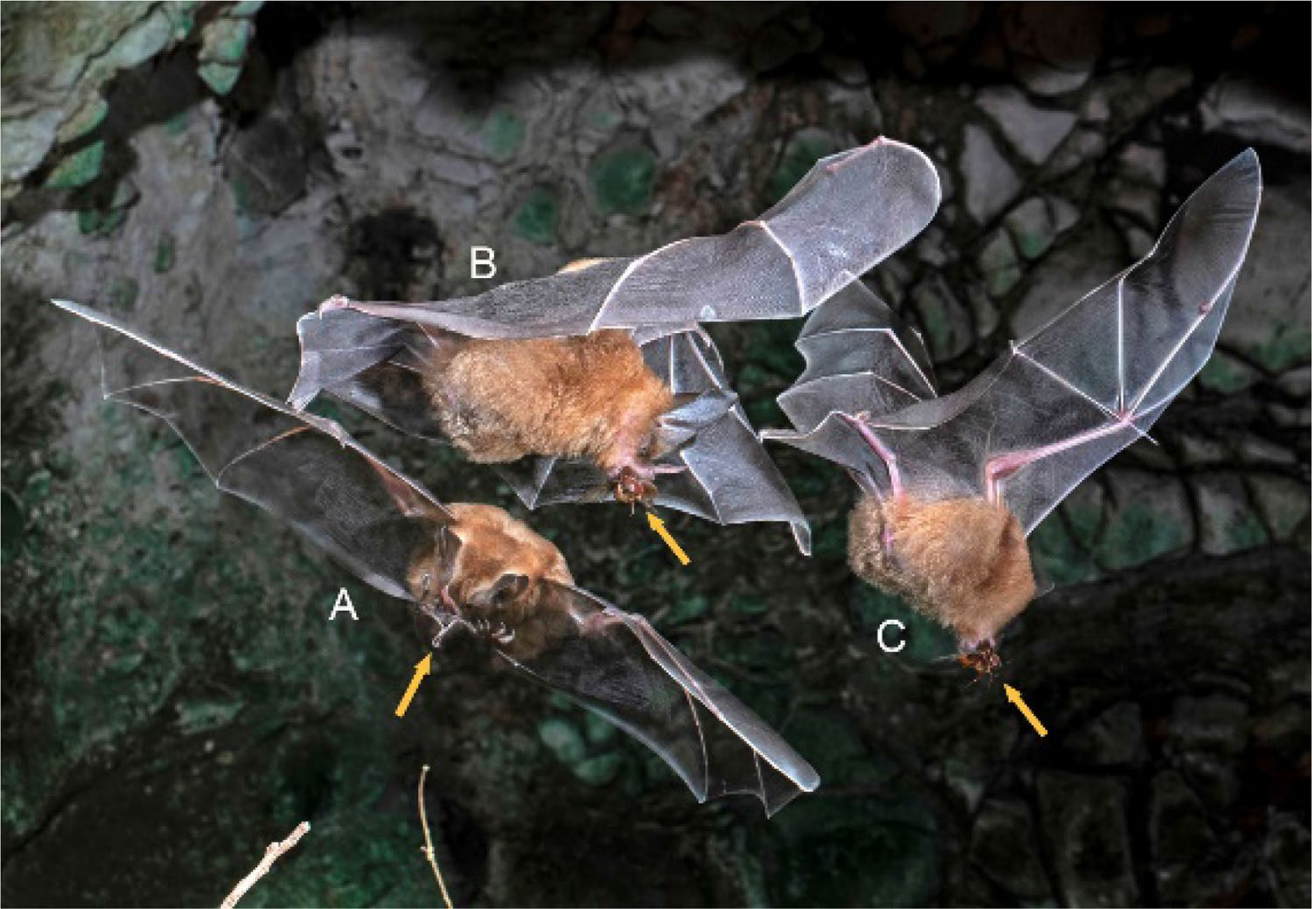
Occasionally, photographs show bats actively handling prey. In this case, three views of the same golden bat (*Mimon cozumelae*) were captured on one frame when it tripped the beam repeatedly. The bat had caught a beetle (marked with arrows) in its interfemoral membrane (**A**), which it then transferred to its mouth (**B**, **C**). Nikon D850, 50 mm Sigma art, f/16, bulb, ISO 250. The picture was taken at the entrance to a cave roost in Belize. Photo by S. L. Fenton and M. B. Fenton

**Fig. A2:**
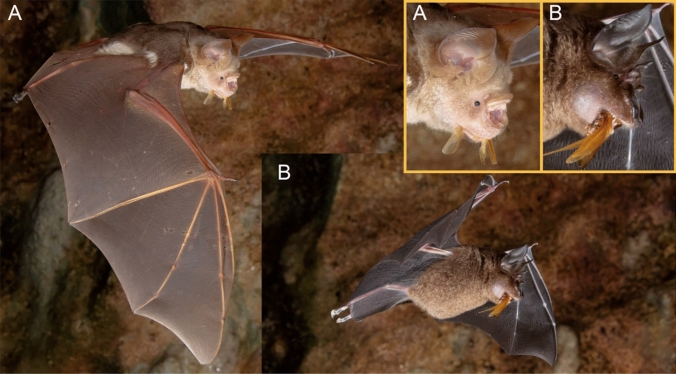
Diadem roundleaf bat (*Hipposideros diadema*, **A**) and Creagh’s horseshoe bat (*Rhinolophus creaghi*, **B**) returning to Gomantong Caves, Sabah in the early morning, 16 min apart. Both individuals have clearly just finished feeding on a swarm of the same species of insects, and have returned to the cave without finishing chewing them. The diadem roundleaf bat is readily recognized by its large size and the distinctive white stripes down the side of the body. Creagh’s horseshoe bat can be identified by bristles in the middle of the noseleaf instead of a connecting process. Nikon D7200 with Nikon 18–200 mm zoom lens at 52 mm, f/14, bulb, ISO 400). Photos by C. M. Francis

**Fig. A3:**
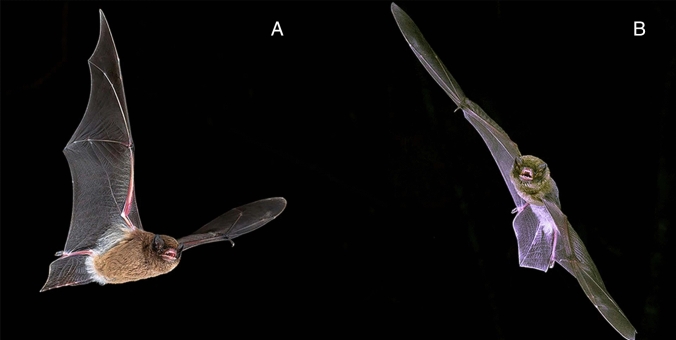
Two Natal long-fingered bats (*Miniopterus natalensis*) at a cave in South Africa. Bats in this genus are easily identified by the last finger bone (phalanx) being nearly 3× longer than the next bone. The color differences are associated with an annual molt. Adult with completed molt (**A**) and younger bat molting from juvenile gray fur to adult brown fur (**B**). **A** Canon 7D, 85 mm lens, f/16, bulb ISO 400; **B** Canon 7D, EF 100 mm lens, f/16, bulb, ISO 400. Photos by ECJ Seamark

**Fig. A4:**
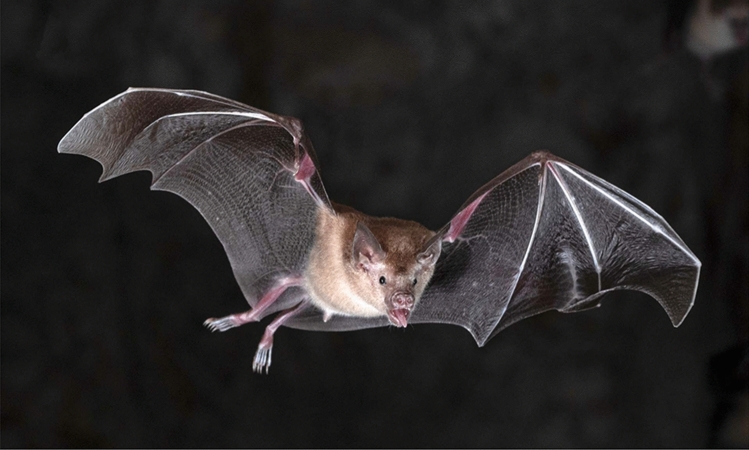
Bats sometimes have distinctive markings providing the opportunity to identify individuals. Here a brown flower bat (*Erophylla sezekorni*) in Jamaica with a distinctive scar on its left wing. Nikon D850, 150 mm Sigma macro, f/20, bulb, ISO 320. Photo by S. L. Fenton and M. B. Fenton

**Fig. A5:**
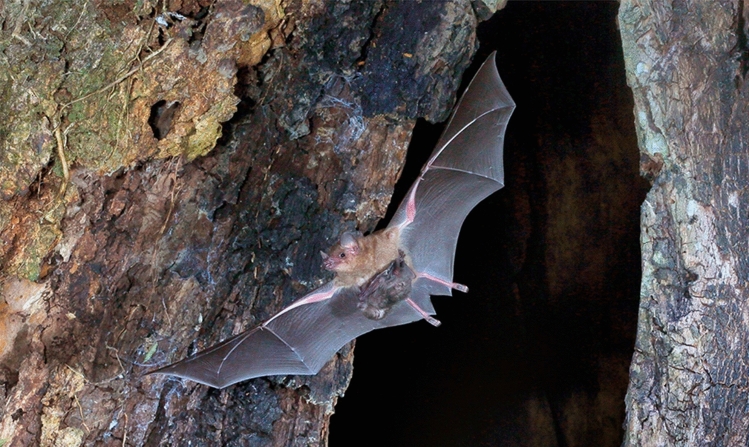
Photography can be used to determine the reproductive status of bats in a roosting site. In this case, the photograph revealed a mother Pallas’s long-tongued bat (*Glossophaga soricina*) leaving a tree roost with her young. Canon 5D Mk III with Canon 50 mm lens, f/16, bulb, ISO 250. Photo by J. Rydell (uncropped version in Supplementary material 1)

Photographs can provide other information about the behavior and ecology of bats such as feeding behavior (Fig. 7 and Fig. A2 in Appendix), or timing of emergence from roosts. Pattern and timing of molt is evident in some pictures (Fig. A3 in Appendix). Wing scars can be distinctive and make it possible to recognize individuals (Fig. A4 in Appendix), or to determine whether bats may show signs of disease such as White Nose Syndrome. Timing or state of reproduction can also sometimes be determined, such as when females are lactating (Fig. S2 in Supplementary Material No. 1), pregnant, or carrying young (Fig. A5 in Appendix and Fig. S3, S4, S11 in Supplementary Material No. 1).

### Choosing places to photograph bats

A good place to photograph bats is one where they congregate to roost, to feed, or to drink, and where their flight paths are relatively predictable. For example, on nights in August and September, many north temperate zone bats ‘swarm’ (Fenton 1969; Schaik et al. 2015) at sites they later use for mating and hibernation (Fig. S6 in Supplementary Material No. 1). At swarming sites, photographic sampling can reveal what species of bats use the site without capturing and handling them. Through photographic monitoring, we have learned that bats drink from an astonishing variety of sites, from puddles (Fig. 6), to pools and tanks (Fig. S7, S8 in Supplementary Material 1). Photographic techniques can also be used to determine which nectar feeding bats are visiting flowers or artificial feeders (Fig. S11 and S16 in Supplementary Material 1). When choosing a site to survey in a previously unknown area, information from local biologists or naturalists can be especially helpful for finding good sites—including potentially unexpected locations (e.g., Fig. S4 in Supplementary Material 1). Setting cameras at even well-known sites can often reveal surprises. For example, at several underground sites in Belize, our photographs provided the first indications that fringe-lipped bats (*Trachops cirrhosis*) and Mexican funnel-eared bats (*Natalus mexicanus*—Fig. S9 in Supplementary Material 1) often used the roosts. Several years later, we have yet to catch a Mexican funnel-eared bat in this area in a mist net or harp trap, but we often photograph them emerging from roosts. Similarly, in the same areas in Belize in November 2021 we often photographed golden bats, even though we did not catch them during traditional sampling.

### Obtaining ‘Reference’ photographs of bats

Working with colleagues who are catching bats for other projects can be an opportunity to obtain reference photographs showing details of diagnostic features to help with identification of photographs of free-flying bats, as well as for educational and other purposes.

For close-up shots of a hand-held bat, we have used a macro lens (105 or 150 mm), with two or three flashes set to provide enough light to reveal details when shot at ISO 200 and f/16. We gently restrain the bat so we can see the details we want. We always use a tripod and an electronic or cable shutter release and keep each photo session as short as possible (maximum 10 min). For small bats (< 20 g) hand-held portraits are an effective way of capturing anatomical details that may later be visible in a cropped view of a flying bat. In some cases, it may also be possible to place bats gently on a branch or other substrate and quickly take photos before they fly away. If the photos are taken in a flight tent (see below), the bat can be recaptured if it flies off too early, using a hand net.

**Fig. 8:**
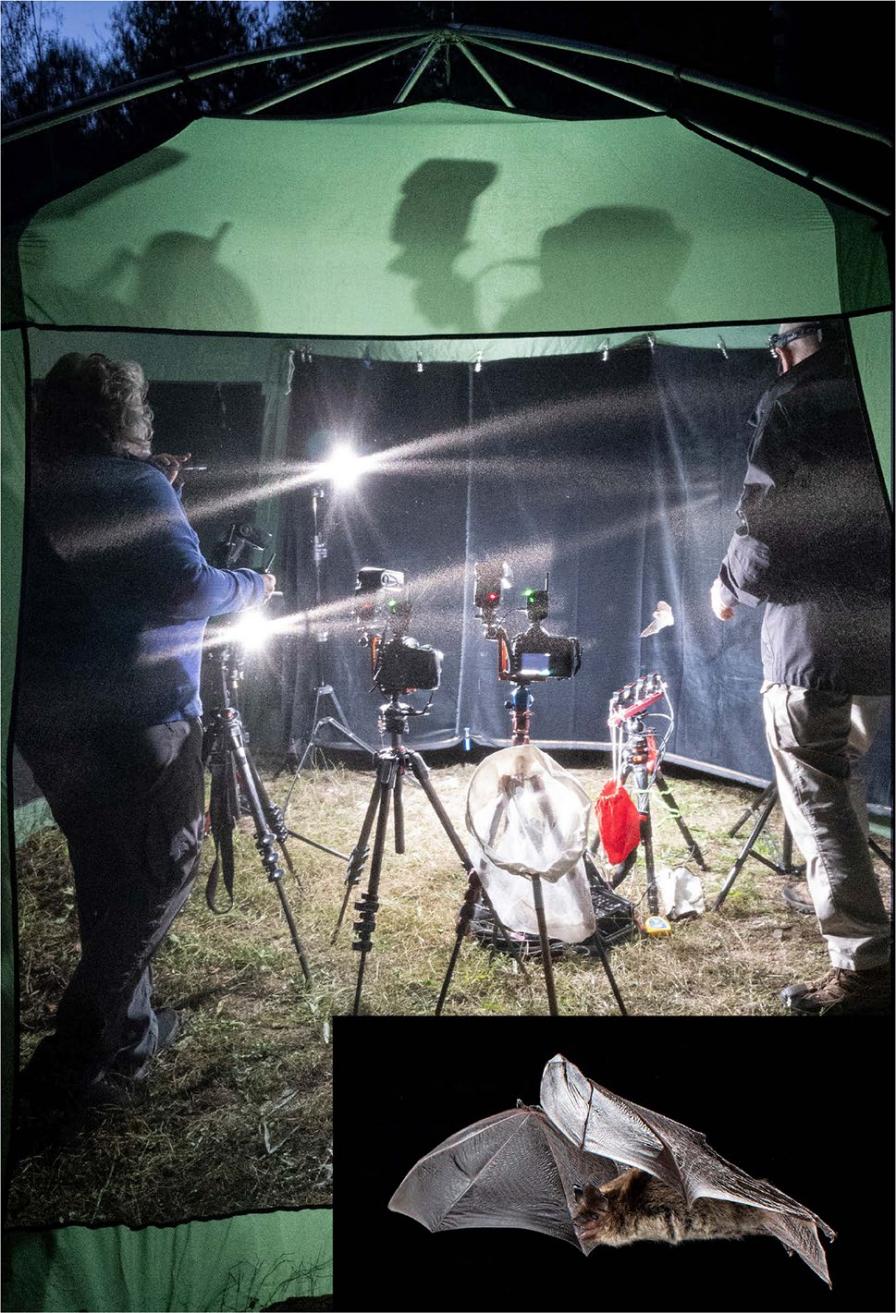
SLF and MBF in a screen tent set up as a studio for photographing flying bats. The tent is 2 m by 4 m by 2.5 m tall. A little brown myotis (*Myotis lucifugus*) can be seen flying just above the four beams. Two of 8 flashes are obvious, as are three cameras each on a tripod. There is a hand net leaning against one tripod for recapturing the bat. A black cloth has been hung from one side of the tent for a background. We had pitched the tent near an abandoned mine used by swarming bats. Tent photo by Lily Hou. Inset photo shows one of the photographs of the little brown myotis taken with this setup by S. L. Fenton and M. B. Fenton

Flying captured bats in a suitably sized tent or room can be an effective way to photograph known species in flight (Fig. 8). Commercial bug shelters with mesh sides, designed to fit over picnic tables, can work well as a flight tent. In this situation, we have used the same beam system(s), supports, cameras, lenses and flashes as for photographing wild bats. By placing black cloth or velvet along one wall of the tent/room, we can create a uniform backdrop to highlight the bats. We use an eyedropper to give the bats water and try to provide mealworms or other insects for insectivorous species or sugar water for nectarivorous or frugivorous species to sustain them.

To release a bat in the enclosure, we use a glove to hold it at waist height in a cupped, gloved hand (bat in the palm of one hand facing the flight direction), with the other hand over top. We then gently move the top hand away. Sometimes the bat will take off immediately, break the beam and take its picture. More often, we encourage the bat to fly by gently tapping the back of the hand holding the bat. We always have a hand net (butterfly net) at the ready to recapture the bat after the flight. We usually release bats after 10–15 flights or attempts.

Not all authors on this paper endorse this use of captured bats, concerned that the process can be too disturbing to the bats.

## Discussion and conclusions

We have shown that photography can be a powerful tool for studying bats that, along with acoustic methods, provides a valuable non-invasive approach for surveying bats. Photographic data can provide important information on bat occurrence as well as behavior (Glaeser et al. 2017). They can be especially useful for detecting species that use soft echolocation calls (e.g., many Phyllostomidae, Nycteridae, Megadermatidae, Kerivoulinae, Murininae, *Plecotus*, and even some *Myotis*) or species that do not echolocate at all (most Pteropodidae). Many of these species often go unnoticed in acoustic surveys but are conspicuous and obvious in photographs.

As with identification of bat species by their calls, photographic surveys may not always allow consistent, accurate identification of all species. The problem of identifying bats by their echolocation calls has received considerable attention (Russo et al. 2018; Fraser et al. 2020), but less attention has been paid to the challenge of identifying flying bats from photographs. In both techniques, the level of challenge of identification varies among situations. For example, in some areas we may already know which bat species to expect at a site, but in other situations (e.g., bats drinking—Rydell and Russo 2014; swarming at hibernacula—Fenton 1969; or surveying sites in previously unsampled regions), the choice of species may be much broader, making identification more challenging. As noted above, in some cases photography and bioacoustics can be combined to enhance identification.

We recognize that some research requires capture and handling wild bats, and we are not suggesting stopping such activities. For example, capture-based surveys are central to understanding the role bats could play in epidemiology (Streicker and Gilbert 2020). Furthermore, many cryptic bat species are difficult to identify conclusively whether in a photograph or in the hand, and may require molecular methods for confident identification (e.g., Francis et al. 2010; Clare et al. 2011), although novel approaches may allow collection of DNA material such as from air in a roost without handling bats (Clare et al. 2022). Neither acoustic nor photographic surveys alone can provide reliable information on population sizes or demographics, which may require additional methods such as mark-recapture studies.

We do argue, however, that capture and handling should be avoided whenever possible, to minimize disturbance and negative impacts on bats associated with handling. Acoustic approaches are rapidly expanding in popularity (e.g., Robinson and Robinson 2021), and here we show how they could be supplemented by photographic surveys.

As a final point, we remark that striking high quality photographs of study animals can be used as promotional materials to make bats and bat ecology more accessible to other citizens, including colleagues, students, or people in naturalists’ clubs.

### Electronic supplementary material

Below is the link to the electronic supplementary material.

Supplementary file1 (PDF 7970 KB)

Supplementary file2 (PDF 206 KB)

## Data Availability

All cited photographs are included in the manuscript or supplementary materials.

## Appendix

See Figs. A1, A2, A3, A4 and A5.
