# Supplementary material for: Bat selfies: photographic surveys of flying bats
Source: Mamm Biol. 2022 Apr 7;102(3):793–809. doi: 10.1007/s42991-022-00233-7 (PMC8988114; doi:10.1007/s42991-022-00233-7)
Supplement: Supplementary file 1 — Supplementary file1 (PDF 7970 KB) [file 42991_2022_233_MOESM1_ESM.pdf]

Supplementary Material No. 1

Bat selfies - Photographic surveys of flying bats

Jens Rydell[1†], Danilo Russo[2], Price Sewell[3], Ernest C. J. Seamark[4], Charles M. Francis[5], Sherri L. Fenton[6], M. Brock Fenton[6*]

1. Department of Biology, Lund University, S-22362 Lund, Sweden. Deceased.

2. Wildlife Research Unit, Dipartimento di Agraria, Università degli Studi di Napoli Federico II, via Università 100, 80055 Portici, Napoli, Italy. danrusso@unina.it

3. Copperhead Environmental Consulting, 471 Main Street, Richmond, Kentucky, USA. psewell@copperheadconsulting.com

4. AfricanBats NPC, 357 Botha Ave, Kloofsig, 0157, South Africa. ernest.seamark@africanbats.org

5. Canadian Wildlife Service, Environment and Climate Change Canada, Ottawa, Ontario, Canada. charles.francis@ec.gc.ca

6. Department of Biology, University of Western Ontario, London, Ontario, Canada. bfenton@uwo.ca

† Deceased

*Corresponding author: Brock Fenton – bfenton@uwo.ca

Figures S1 to S17 - Additional examples of photographs of bats in flight.

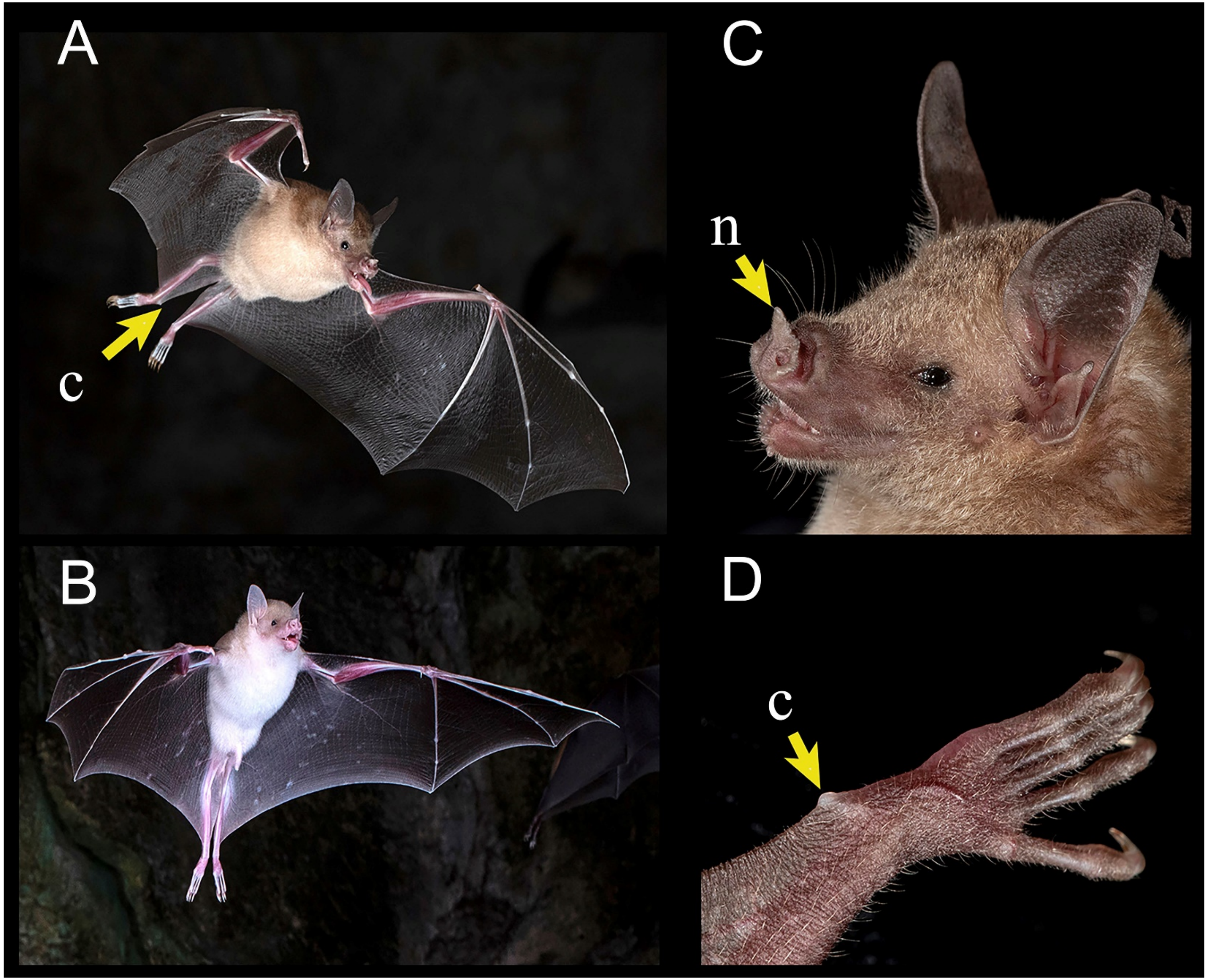

Fig. S1 Brown flower bats *Erophylla sezekorni* (A, D) and Jamaican flower bats *Phyllonycteris aphylla* (B, C) roost in the same cave in Jamaica. The nose-leaf in brown flower bats (B) is much more prominent (arrow from n) than in Jamaican flower bats. Furthermore, the brown flower bat has distinct calcars (c in A and D), which are lacking in the other species (B), and the Jamaican flower bat is whiter and has a thicker penis. Brown flower bat photographed with a Nikon D810, with Nikon 60 mm macro lens, f/16, bulb, ISO 400. Jamaican flower bat with Nikon D850, Nikon 50 mm lens, f/16, bulb, ISO 400. Photos by SL Fenton and MB Fenton.

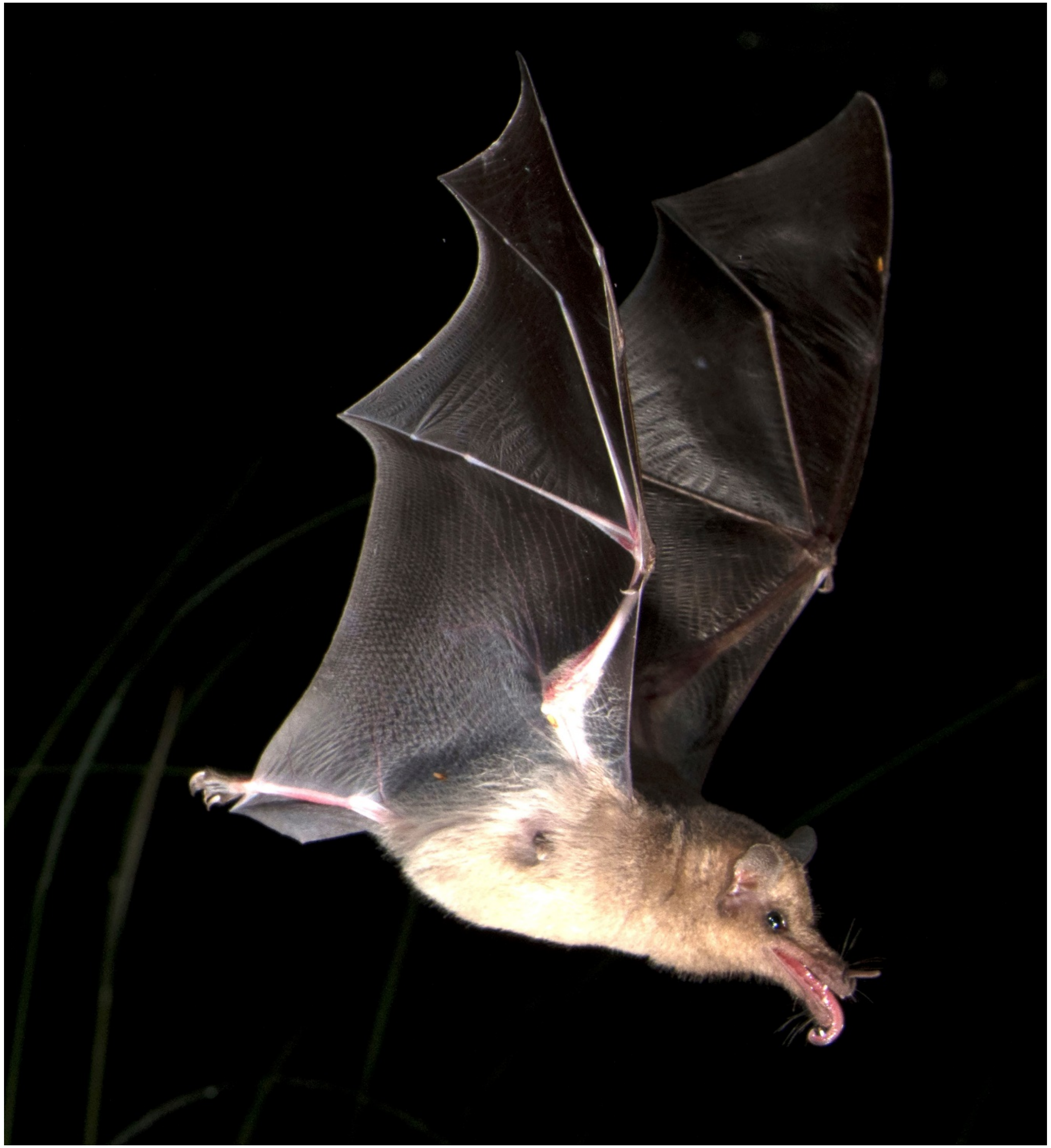

Fig. S2 The nipples of females that are or have been lactating are often obvious in photographs. In this case, a female Mexican long-tongued bat (*Choeronycteris mexicana*) shows clear evidence of having nursed recently. Nikon D850, Nikon 20-200 mm lens (at 70 mm), f/16, 1/250s, ISO 200. Photo by SL Fenton and MB Fenton.

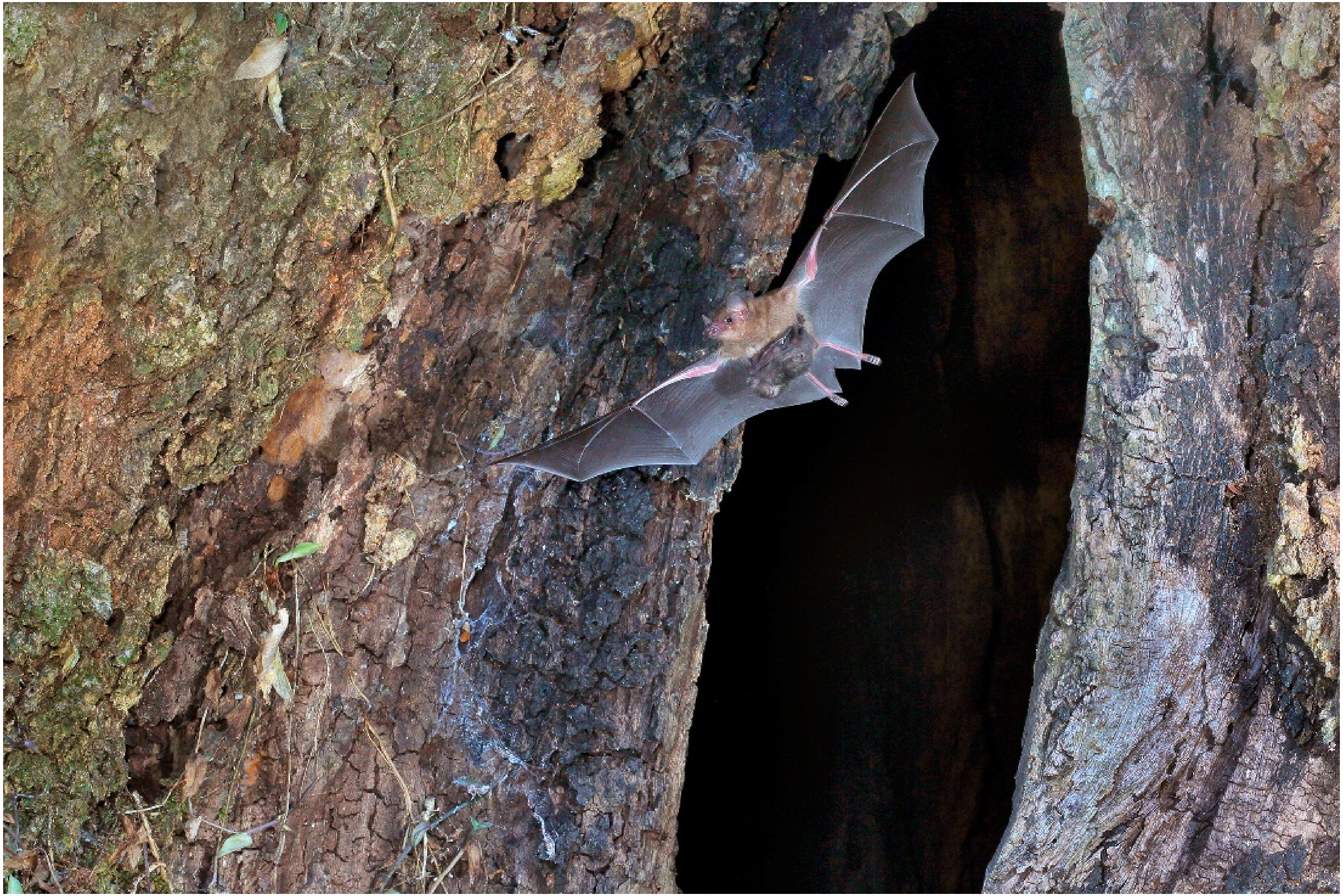

Fig. S3 Photography can be used to determine which species of bats are roosting in a hollow tree, without capturing them. In this case, the photograph revealed a mother Pallas's long-tongued bat (*Glossophaga soricina*) leaving a tree roost with her young. Canon 5D Mk III with 50 mm lens, f/16, bulb, ISO 250. Photo by J Rydell.

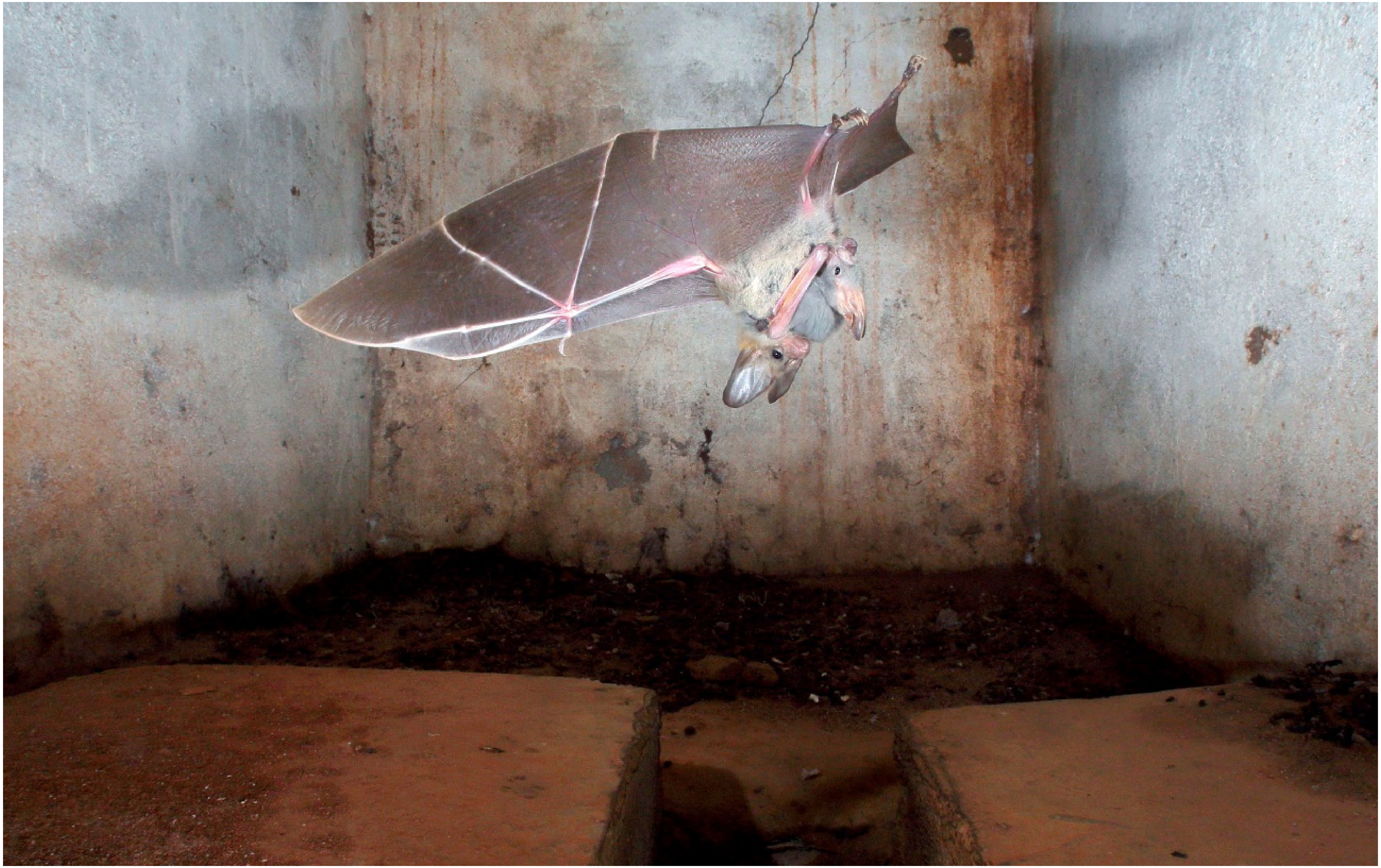

Fig. S4 Camera traps can also be set in unusual locations such as this pit toilet in Africa, which had a small maternity colony of heart-nosed false-vampires (*Cardioderma cor*) roosting in the pit, as revealed by this photograph of an adult female carrying its young. Canon 5D Mark III with 14 mm lens, f/18, bulb, ISO 320. Photo by J Rydell.

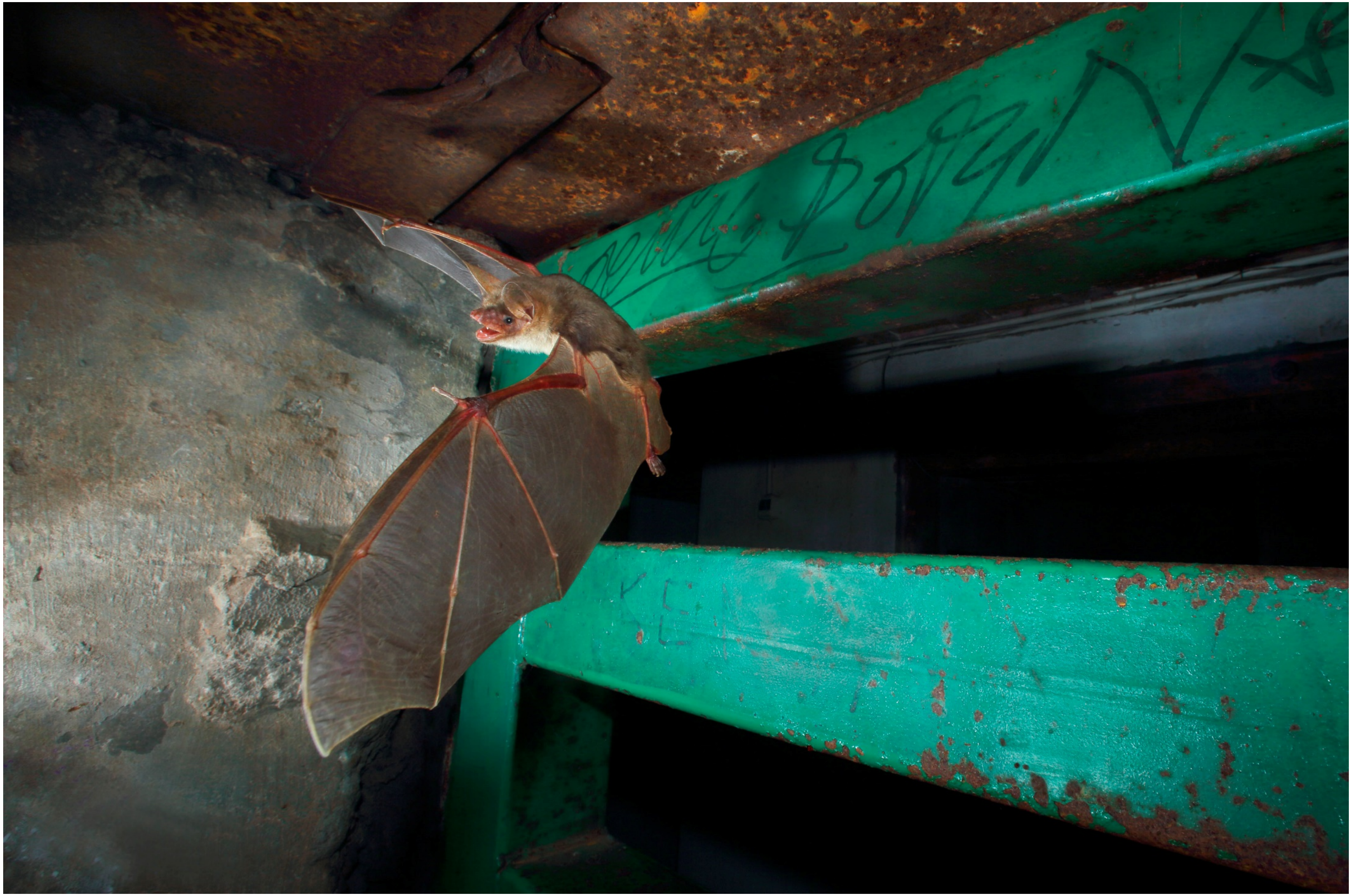

Fig. S5 A greater mouse-eared bat (*Myotis myotis*) flying out of a roost in a former Nazi bunker in Nietoperek, Poland. Canon 5D Mark III with 14 mm lens, f-16, bulb, ISO 160. Photo by J Rydell.

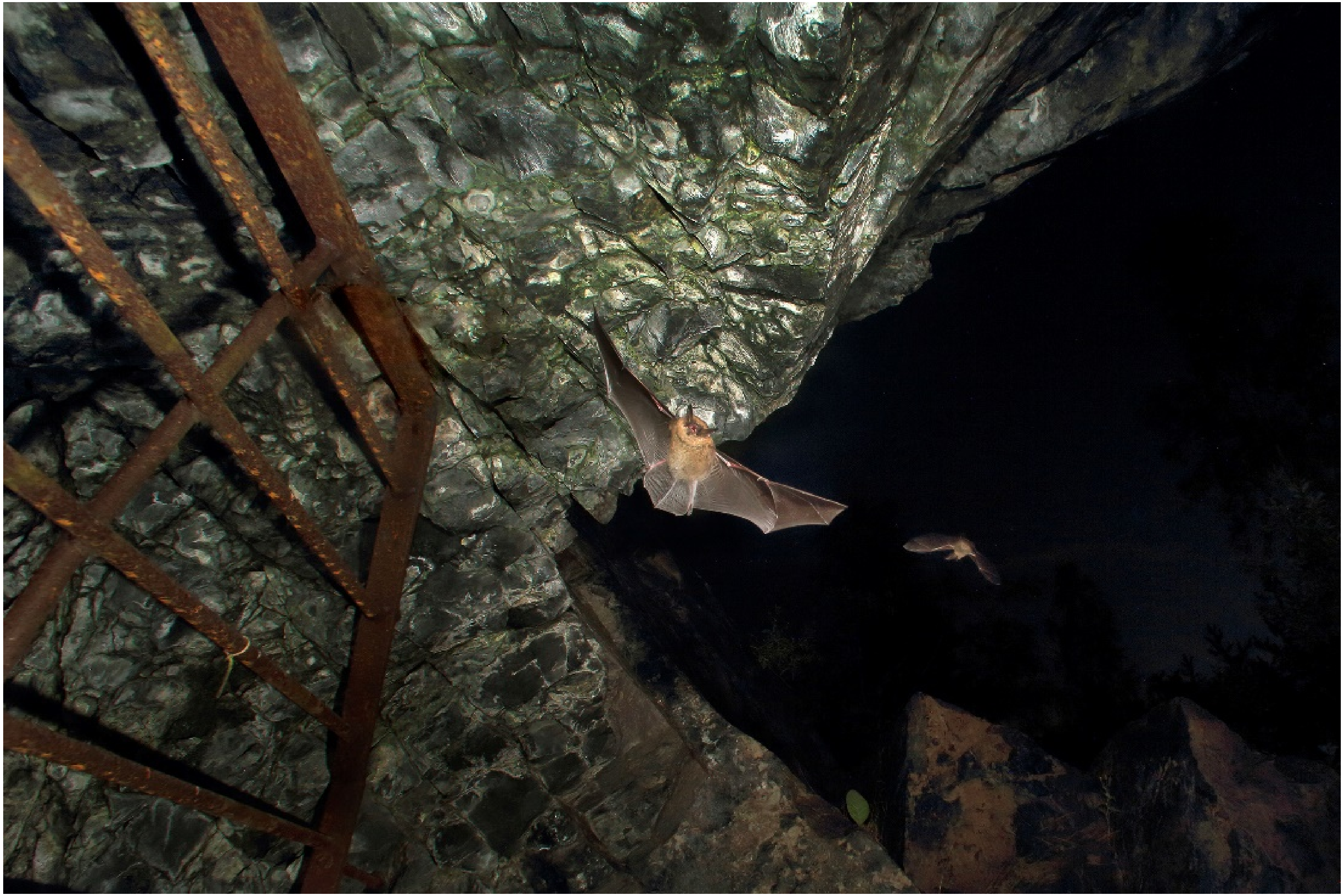

Fig. S6 Bats swarming at the entrance to a mine. The closest bat is either Brandt's myotis (*Myotis brandtii*) or whiskered myotis (*M. mystacinus*), the dark face suggesting the latter. A brown long-eared bat (*Plecotus auritus*) is in the background, easily recognized by its huge ears. Canon 5D Mark III with 14 mm lens, f/13, bulb, ISO 800. Photo by J Rydell.

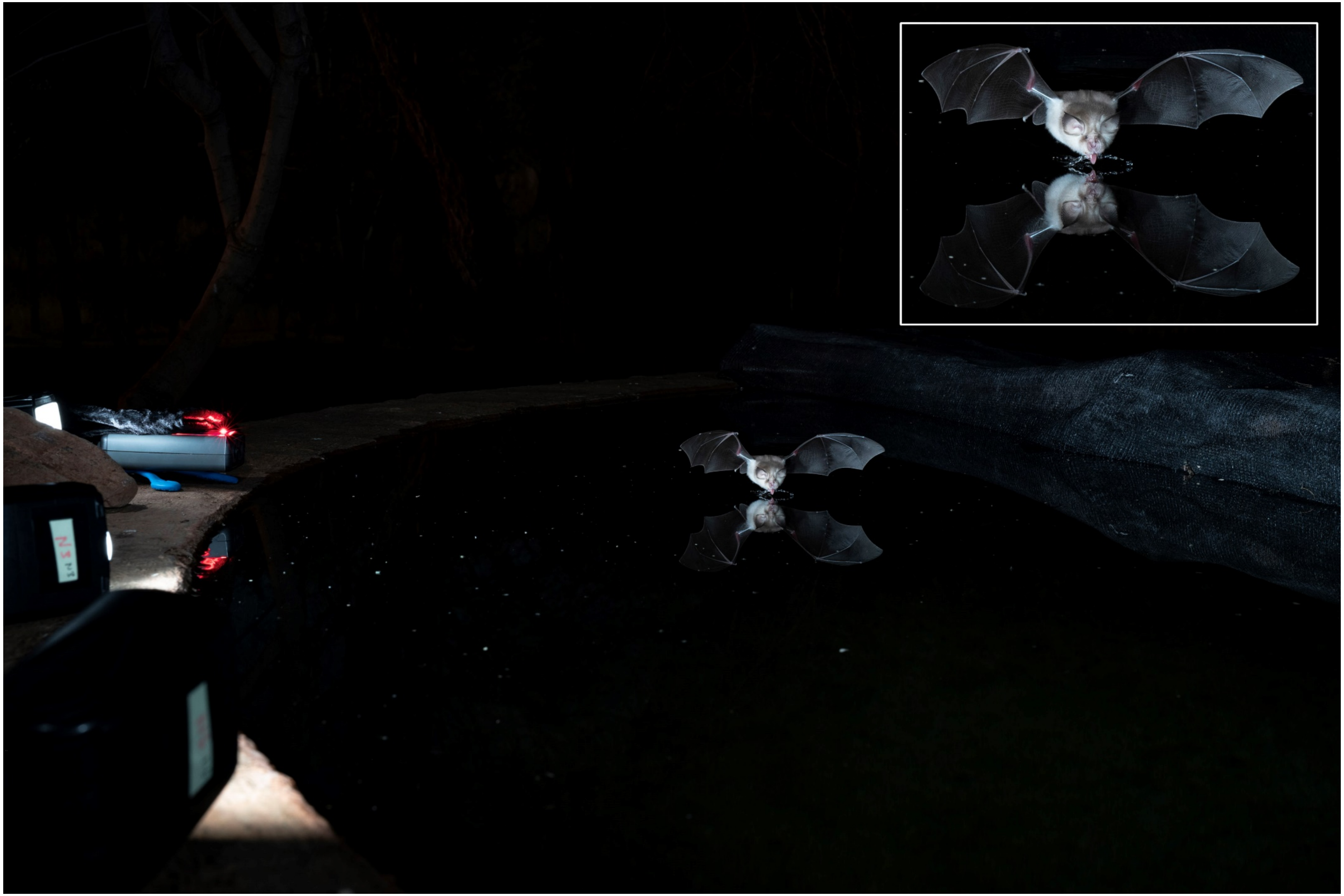

Fig. S7 Photography at ponds and waterholes can be particularly productive in arid areas. This Bushveld horseshoe bat (*Rhinolophus simulator*) was drinking from a tank in South Africa. Part of the pond (to the right) was covered to increase the chance that the bats would fly within reach of the camera. The trigger and two of the flashes can be seen on the left. Nikon D850, Sigma 150 mm macro lens f/16, bulb, ISO 250. Photo by ECJ Seamark, SL Fenton and MB Fenton.

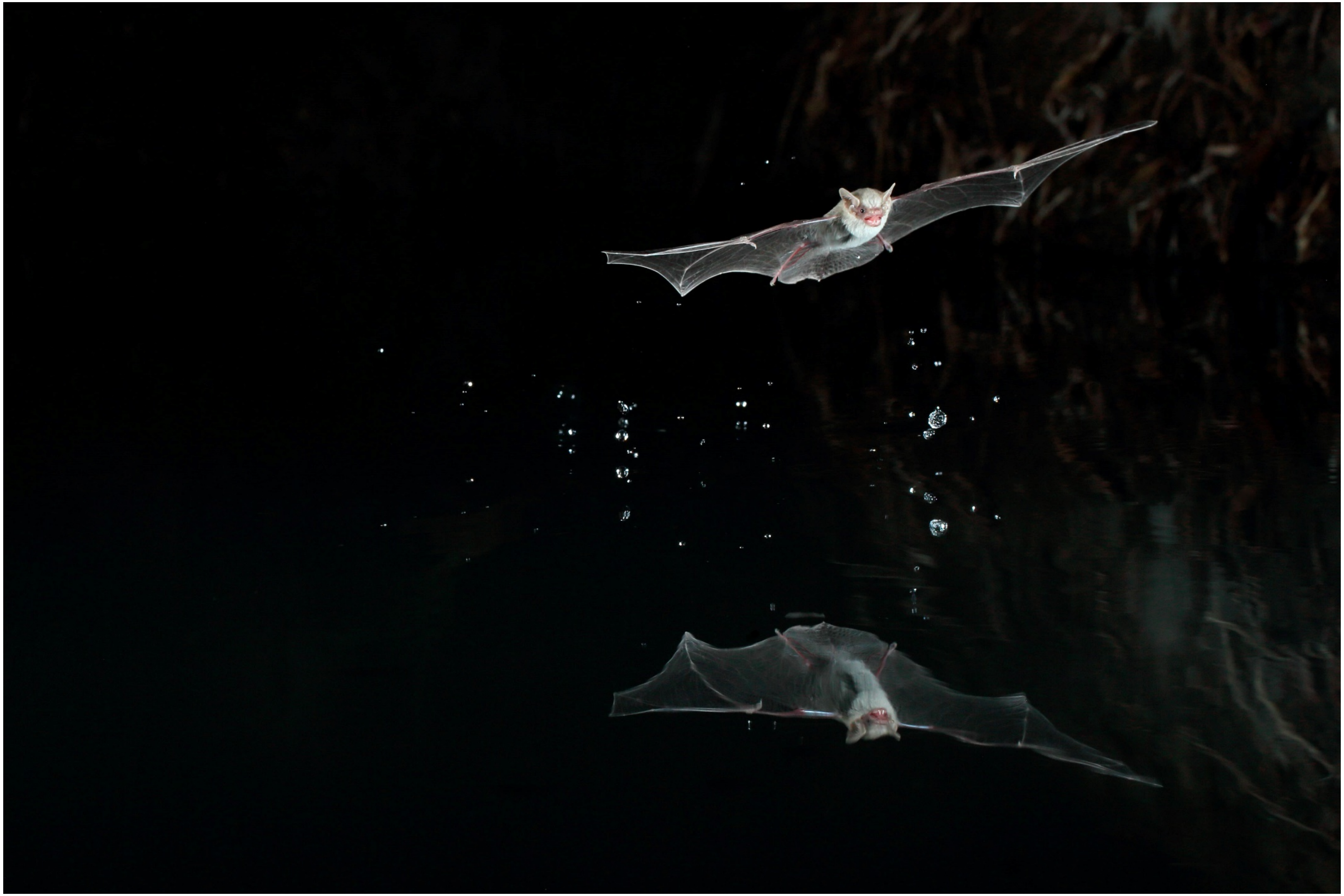

Fig. S8 Another example of a bat photographed drinking, in this case a Bodenheimer's pipistrelle (*Hypsugo bodenheimeri*) at a desert pond in Israel. Canon 5D Mark III with 100 mm lens, f/16, bulb, ISO 400. Photo by J Rydell.

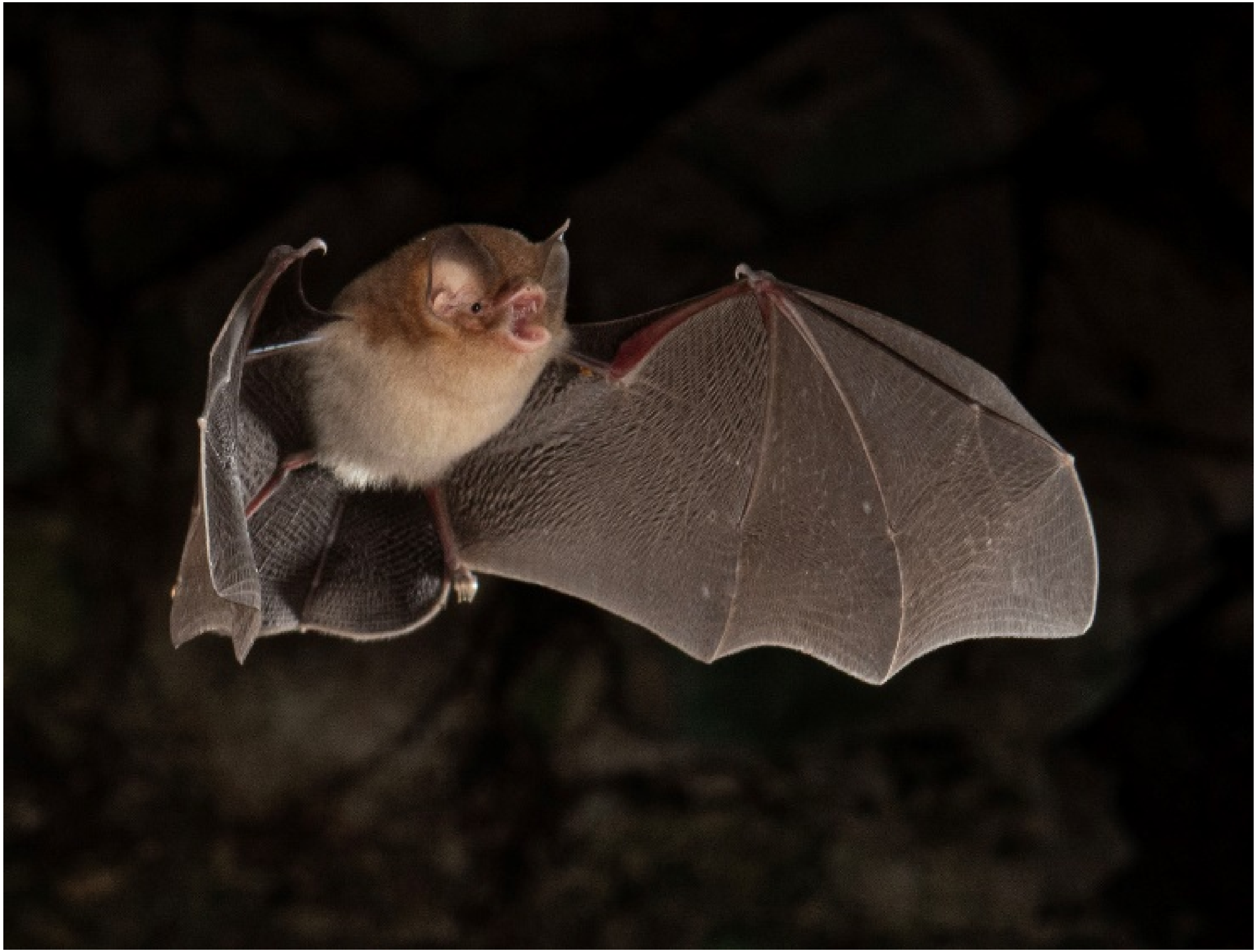

Fig. S9 At some underground sites in Belize we have photographed Mexican funnel-eared bats (*Natalus mexicanus*), but we have not yet netted or trapped one in the area. Nikon D850, 50 mm Sigma art, f/16, bulb, ISO 320. Photo by SL Fenton and MB Fenton.

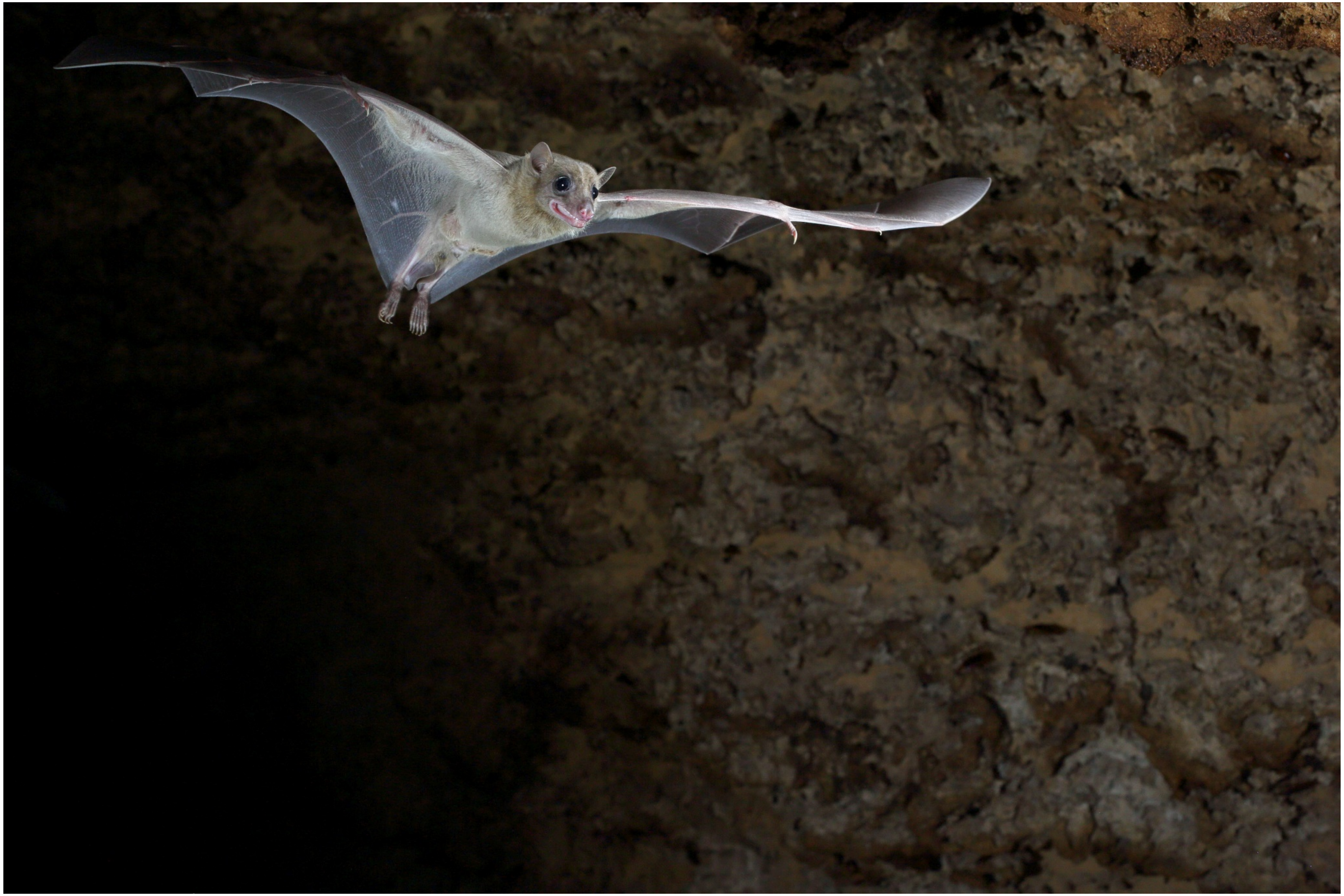

Fig. S10 The genus *Rousettus* is the only group of Old World fruit bats (Pteropodidae) known to echolocate, so could potentially be surveyed acoustically. In photographs, they can be identified by the distinctive shape of their head with a moderately elongate muzzle. This Egyptian fruit bat (*Rousettus aegyptiacus*) photographed leaving a cave is the only member of the genus that occurs in Africa. Canon 5D Mk III with 100 mm lens, f/13, bulb, ISO 320. Photo by J Rydell.

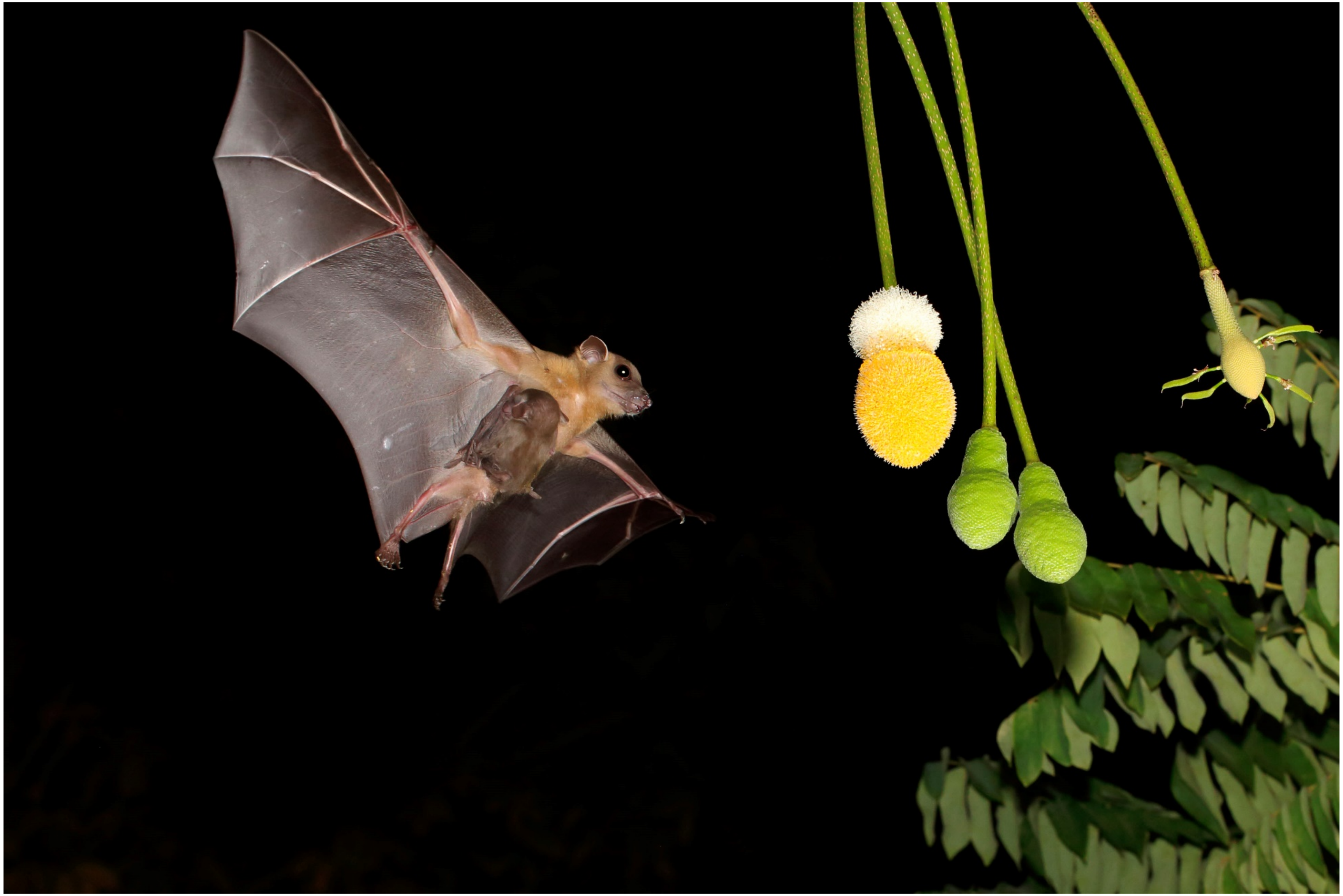

Fig. S11 Bats are important pollinators of several commercially important tree species, including durian (*Durio* spp.) and petai (*Parkia speciosa*). Photography can be a useful way to determine which species of bats are visiting the flowers. In this case, a lesser short-nosed fruit bat (*Cynopterus brachyotis*) was visiting a petai flower in Thailand while carrying its baby. Canon EOS 5D Mk III with 100 mm lens, f/13, 1/125, ISO 640. Photo triggered by hand by J Rydell.

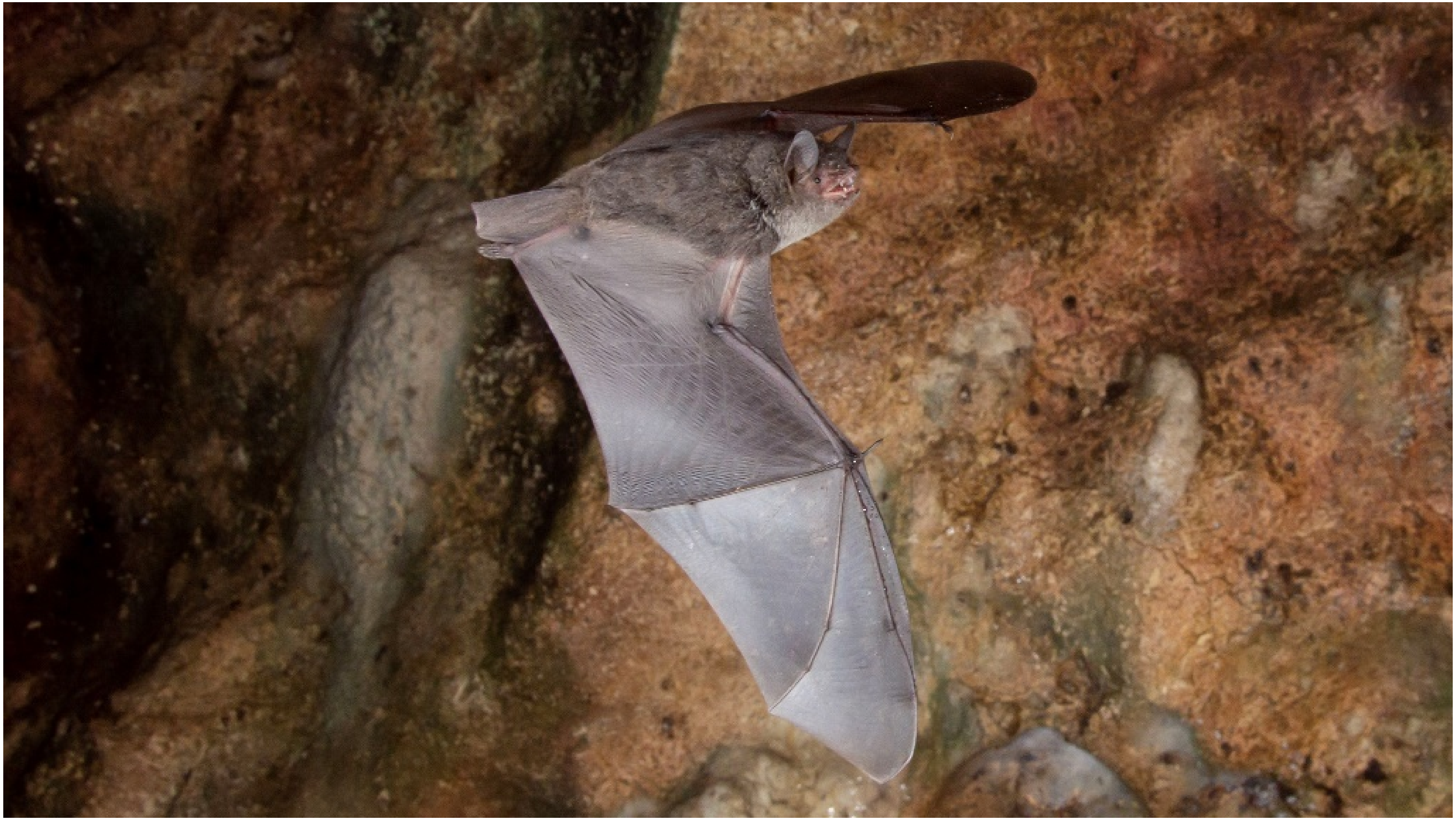

Fig. S12 Horsfield's myotis (*Myotis horsfieldi*), here seen returning in the early morning to Gomantong Caves, Sabah, can be distinguished by slightly enlarged feet with the wing membrane attached to the side of the foot, half way between the ankle and the base of the toe. Nikon D7200 with Zoom lens set at 29mm focal length, f/14, ISO 400. On bulb with beam triggering flash. Photo by CM Francis.

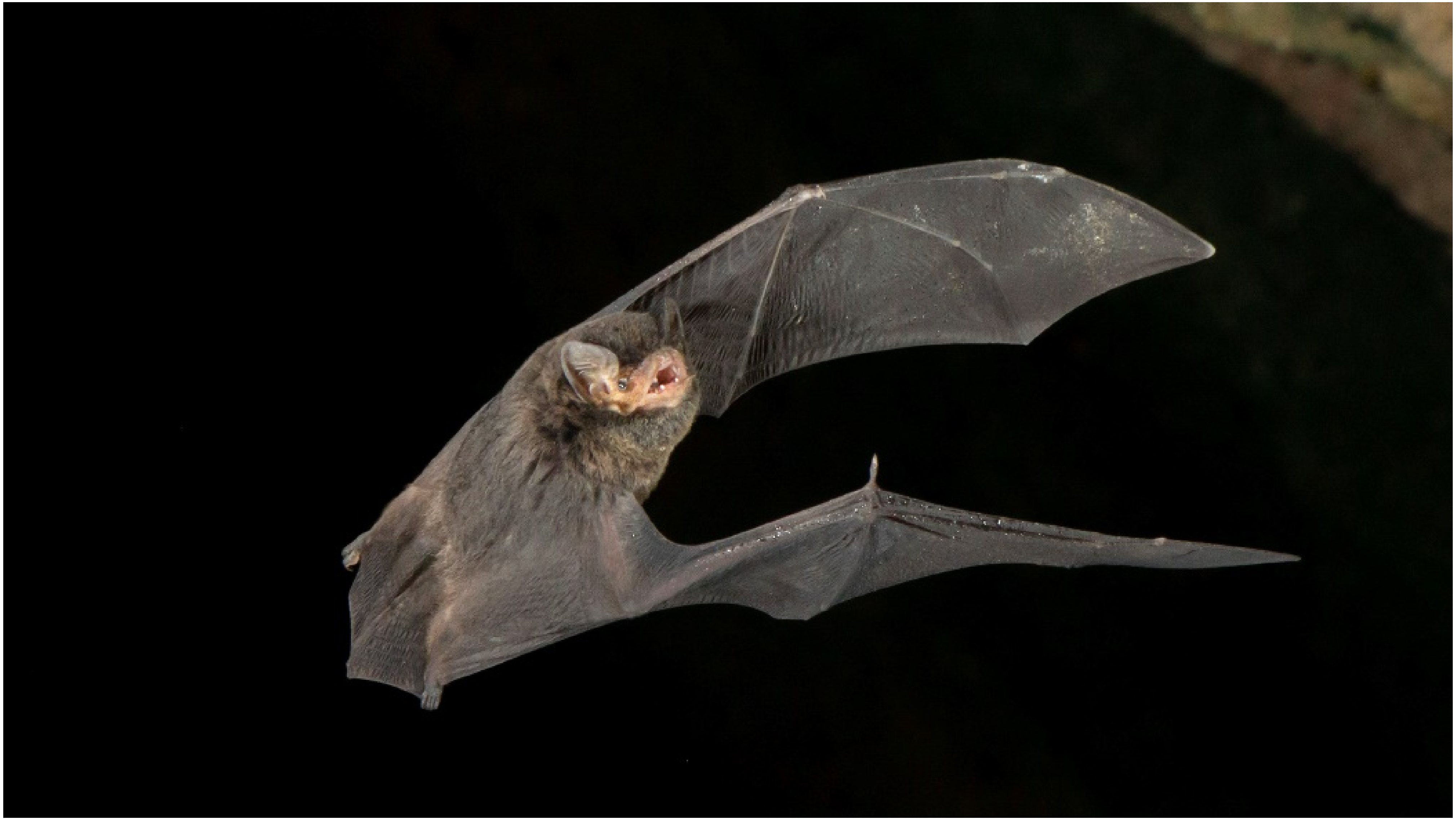

Fig. S13 Bent-winged bats (*Miniopterus* spp.) are readily identified to genus by the shape of the ear and the terminal bone on the longest finger being nearly 3x longer than the next bone. However, in areas where multiple species occur, they can only reliably be separated by measurements or DNA. Photographing a known scale pattern at the focal point may be a useful way to estimate the size of bats in photographs. This relatively small individual Gomantong Caves, Sabah was identified as the lesser bent-winged bat, (*Miniopterus australis*). Canon 7D Mark II, 95mm lens, f/16, ISO 400. On bulb with beam triggering flash. Photo by CM Francis.

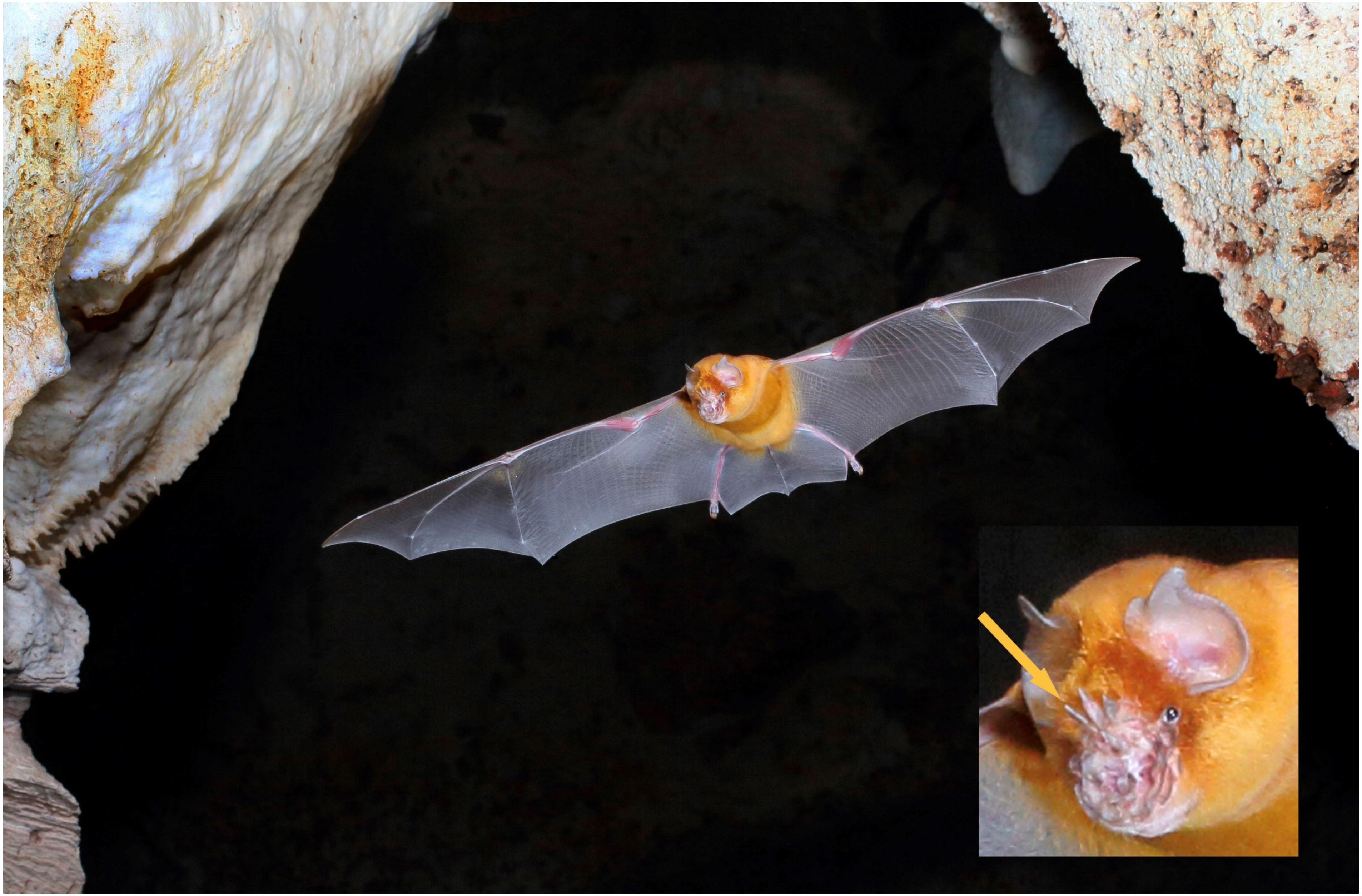

Fig. S14 This African trident bat (*Triaenops afer*) taken at a coastal cave in Kenya is another rmember of the family Rhinonycteridae with a distinctive noseleaf that has 3 tall points (see inset). Recent genetic studies suggest that the populations in the Rift valley in the interior of Kenya are a distinct species, but it is unclear whether they could be separated from a photograph. Canon EOS 5D Mark III, 50 mm lens, f/16, bulb, ISO 500. Photo by J Rydell.

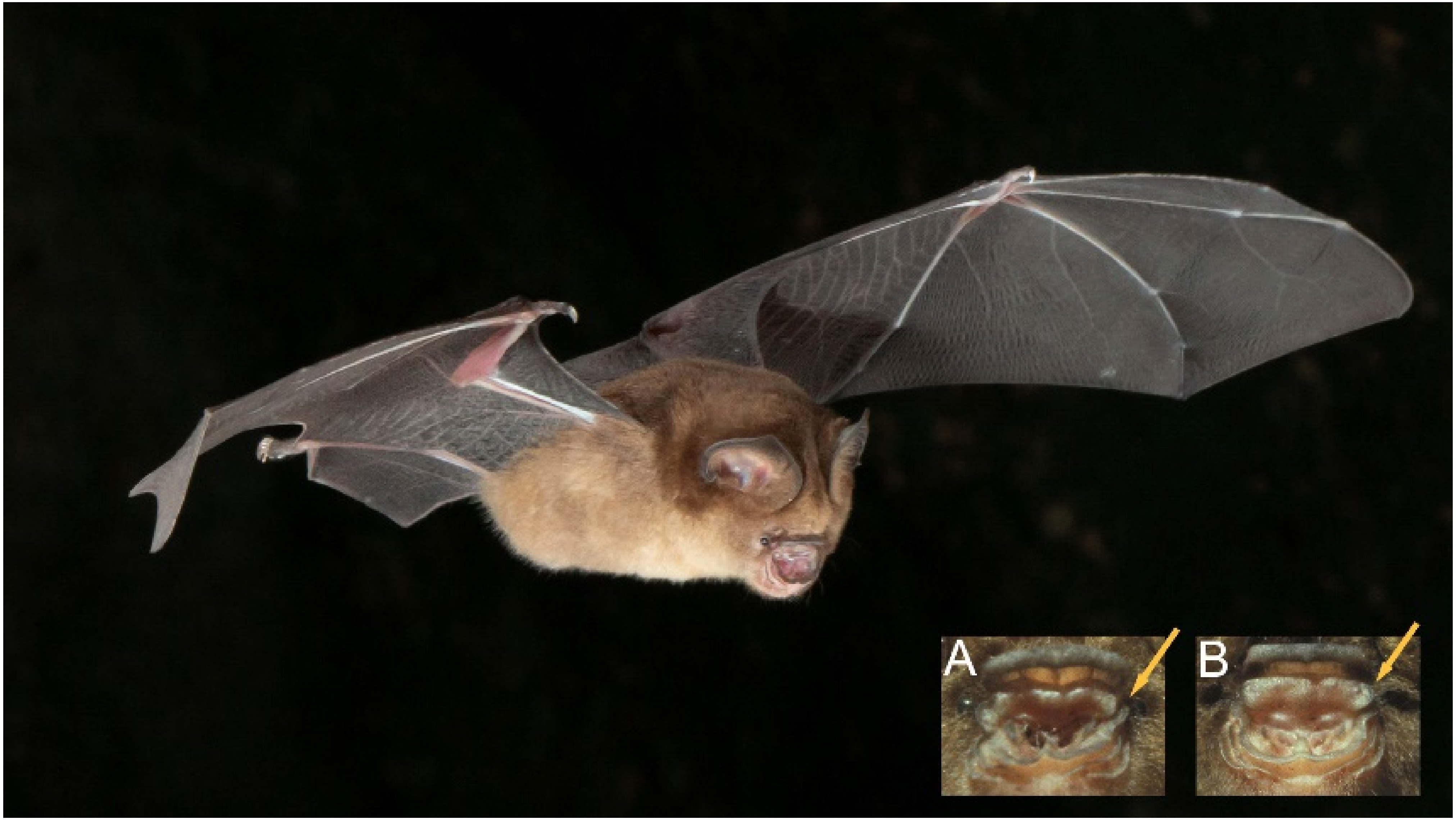

Fig. S15 The fawn roundleaf bat (*Hipposideros cervinus*), is one of two similar species of small *Hipposideros* found in Sabah, Malaysia with two small lateral lappets (flaps of skin) on each side of the noseleaf. They can be distinguished by the shape of the noseleaf which has the median noseleaf (arrows) narrower than the posterior noseleaf in the fawn roundleaf bat (A), but wider than the posterior noseleaf in Cantor's roundleaf bat (*Hipposideros galeritus*, B). Flight photo Canon 7D Mark II, 72mm focal length, f/16, ISO 400. On bulb with beam triggering flash. Main photo and inset reference photos by CM Francis.

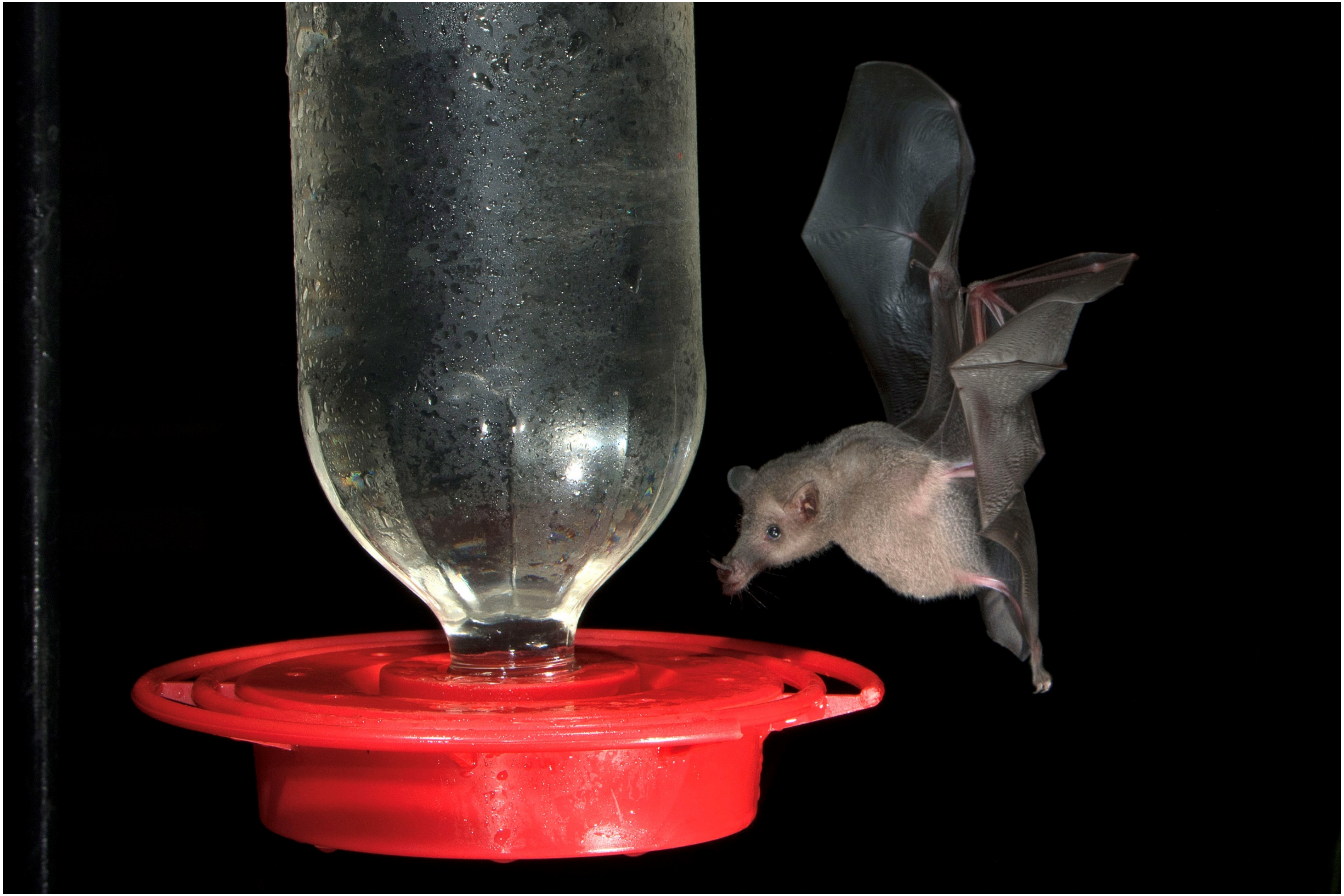

Fig. S16 Some species of nectar feeding bats will come to sugar water feeders, such as those commonly used to attract hummingbirds, which can be a useful opportunity to determine which species are in an area. This Mexican long-tongued bat (*Choeronycteris mexicana*) was photographed at a hummingbird feeder near Portal, Arizona. Nikon D810 with Nikon 70 mm lens, f/16, 1/250, ISO 200, beam triggering camera. Photo by SB and MB Fenton.

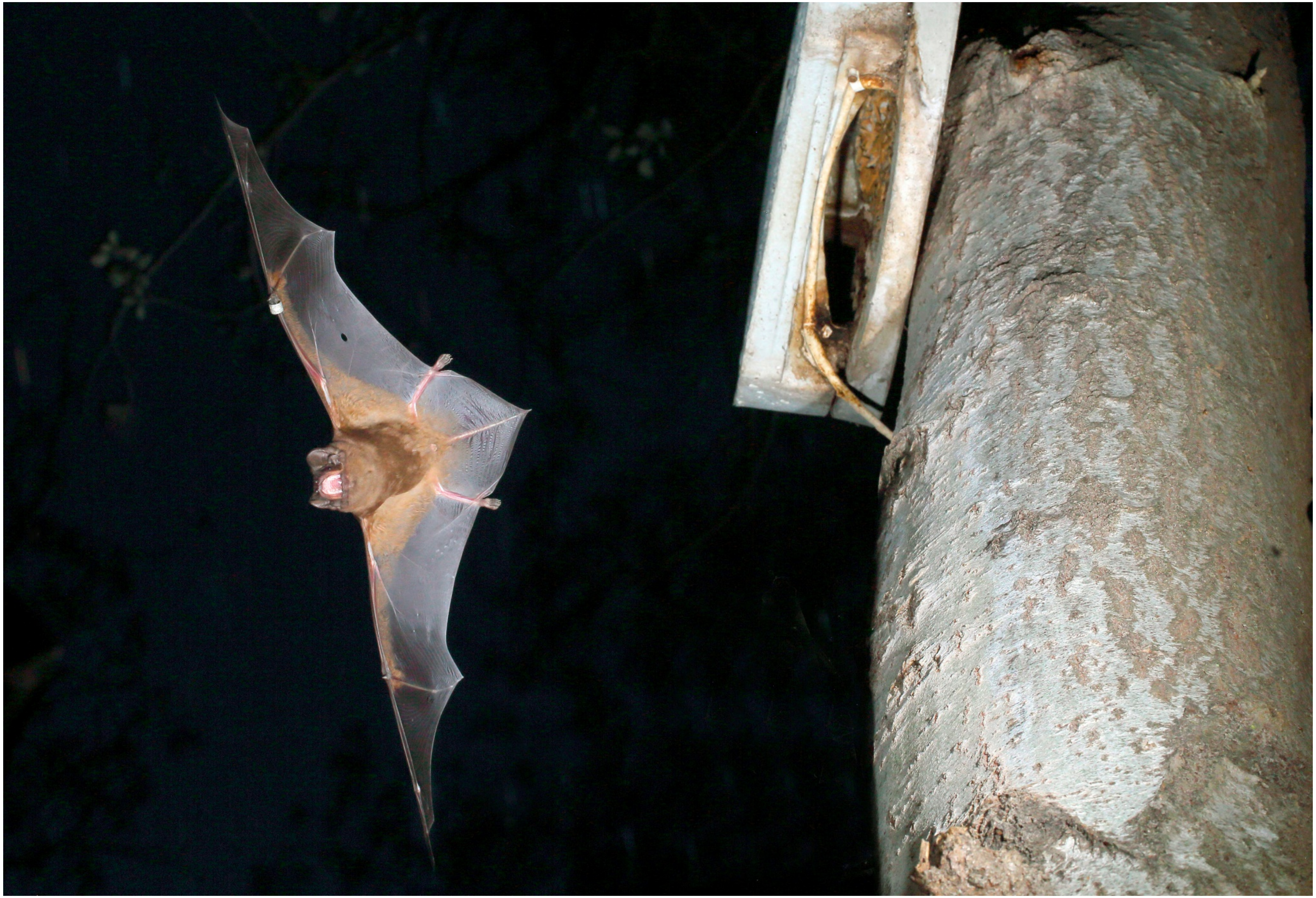

Fig. S17 A greater noctule (*Nyctalus lasiopterus*) emerging from an artificial roost in Doñana National Park, Spain. This bat was banded on the left wing as part of a study looking at dispersal dynamics of the species, but it is not possible to read the full band number in the photo. Canon 5D Mark III with 100 mm lens, f/16, bulb, ISO 400. Photo by J Rydell.
