# Supplementary material for: Bat selfies: photographic surveys of flying bats
Source: Mamm Biol. 2022 Apr 7;102(3):793–809. doi: 10.1007/s42991-022-00233-7 (PMC8988114; doi:10.1007/s42991-022-00233-7)
Supplement: Supplementary file 2 — Supplementary file2 (PDF 206 KB) [file 42991_2022_233_MOESM2_ESM.pdf]

**Supplementary Material no. 2**

# Bat selfies: Photographic surveys of flying bats

Jens Rydell, Danilo Russo, Price Sewell, Ernest C. J. Seamark, Charles M. Francis, Sherri L. Fenton, M. Brock Fenton*

* bfenton@uwo.ca

## Highlights:

- We show how photographic methods can be used to survey flying bats to determine their distribution, habitat use, and roosting sites.
- We describe the types of camera systems that are required, using high-speed flashes and automated infrared trip beam systems, to get sharp, well-focussed photographs that enable species identification.
- Camera traps can be set in areas where bats concentrate in flight, such as exiting roosts in caves, buildings or hollow trees, or drinking over ponds.
- Photographic surveys can complement echolocation call surveys as a non-invasive study method to reduce risks of disturbance or disease transmission in situations where capturing bats is not desirable or necessary.

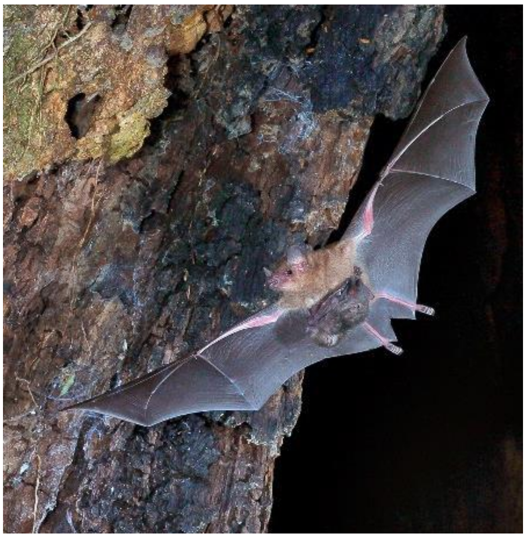

Pallas's long-tongued bat (*Glossophaga soricina*) leaving a tree roost with her young. Photo: Jens Rydell

This article is part of a thematic collection of articles (Special Issue) of *Mammalian Biology* and covers the following topics and taxa (marked with ☑) addressed in the Special Issue:

**Article Type**

| | | | | |
|---|---|---|---|---|
| ☐ Original Research | ☑ Techniques | ☐ Review | ☐ Short Communication | ☐ Concept Note |

**Taxon**

***Terrestrial***

| | |
|---|---|
| ☑ Bats *(Order Chiroptera)* | ☐ Primates : Great Apes *(Family Hominidae)* |
| ☐ Carnivores : Bears *(Family Ursidae)* | ☐ Primates : Old World monkeys *(Family Cercopithecidae)* |
| ☐ Carnivores : Canids *(Family Canidae)* | ☐ Ungulates : Bovids *(Family Bovidae)* |
| ☐ Carnivores : Felids *(Family Felidae)* | ☐ Ungulates : Deers *(Family Cervidae)* |
| ☐ Carnivores : Hyenas *(Family Hyaenidae)* | ☐ Ungulates : Giraffes *(Family Giraffidae)* |
| ☐ Carnivores : Mustelids *(Family Mustelidae)* | ☐ Ungulates : Horses *(Family Equidae)* |
| ☐ Elephants *(Family Elephantidae)* | ☐ Multiple taxa *(3 or more Families/Orders)* |

***Marine***

| | |
|---|---|
| ☐ Baleen whales : Right whales *(Family Balaenidae)* | ☐ Large toothed whales *(Families Delphinidae & Hyperoodontidae)* |
| ☐ Baleen whales : Rorquals *(Family Balaenopteridae)* | ☐ Pinnipeds : True seals *(Family Phocidae)* |
| ☐ Carnivores : Bears *(Family Ursidae)* | ☐ Porpoises *(Family Phocoenidae)* |
| ☐ Carnivores : Mustelids *(Family Mustelidae)* | ☐ Sirenians : Manatees *(Family Trichechidae)* |
| ☐ Dolphins *(Family Delphinidae)* | ☐ Multiple taxa *(3 or more Families/Orders)* |

**Topic**

| | |
|---|---|
| ☐ Acoustic ID | ☑ Identification techniques |
| ☐ Aerial surveys | ☐ Life-history |
| ☐ Analytical innovations | ☐ Machine learning |
| ☐ Automated pattern recognition | ☐ Mark-recapture analysis |
| ☐ Behavioural ecology | ☐ Morphometrics |
| ☑ Camera-trapping | ☐ Network analysis |
| ☐ Conservation management | ☐ Photogrammetry |
| ☐ Data management | ☐ Population ecology |
| ☐ Demographic parameters | ☐ Site fidelity & Movement |
| ☑ Field methodology | ☐ Social ecology |
| ☐ Genetic ID | ☐ Software/Package development |
| ☐ Health conditions | ☐ Thermal imagery |
| ☐ Other: *(please specify)* | |

## References

Karczmarski L, Chan SCY, Rubenstein DI, Chui SYS, Cameron EZ (2022a). Individual identification and photographic techniques in mammalian ecological and behavioural research – Part 1: Methods and concepts. *Mammalian Biology* (Special Issue), 102 (3) https://link.springer.com/journal/42991/volumes-and-issues/102-3

Karczmarski L, Chan SCY, Chui SYS, Cameron EZ (2022b). Individual identification and photographic techniques in mammalian ecological and behavioural research – Part 2: Field studies and applications. *Mammalian Biology* (Special Issue), 102 (4) https://link.springer.com/journal/42991/volumes-and-issues/102-4
